# Supplementary material for: COOKIE-Pro: covalent inhibitor binding kinetics profiling on the proteome scale
Source: Nat Commun. 2025 Sep 30;16:8373. doi: 10.1038/s41467-025-63491-2 (PMC12484723; doi:10.1038/s41467-025-63491-2)

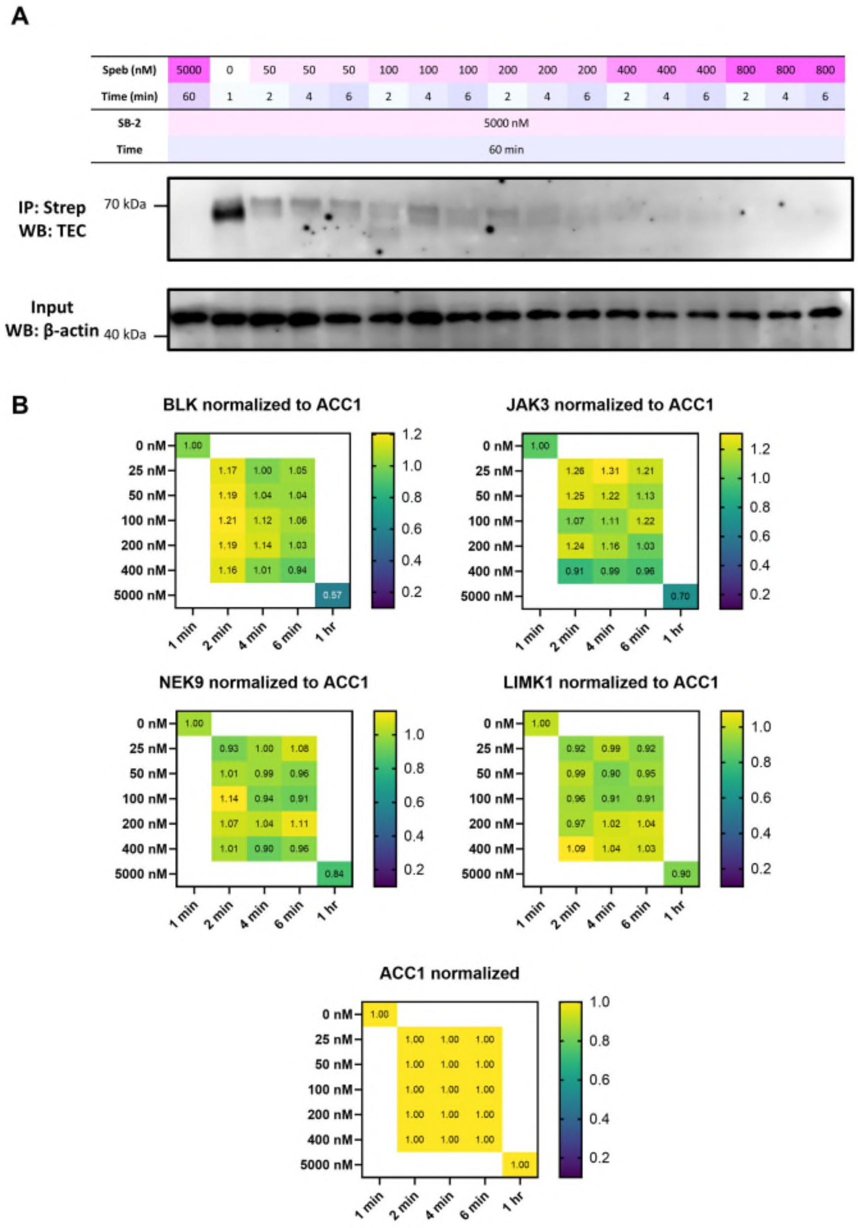

Supplementary Figure 1. Off-targets identification for spebrutinib. (A) Immunoblot of spebrutinib COOKIE samples indicating spebrutinib is a potent TEC kinase binder. Blots are representative for three independent experiments. **(B)** Spebrutinib off-targets that show engagement only at 5 μM treatment for 1 hour. All the data points have been normalized to the acetyl-CoA carboxylase 1 (ACC1) abundance.

A

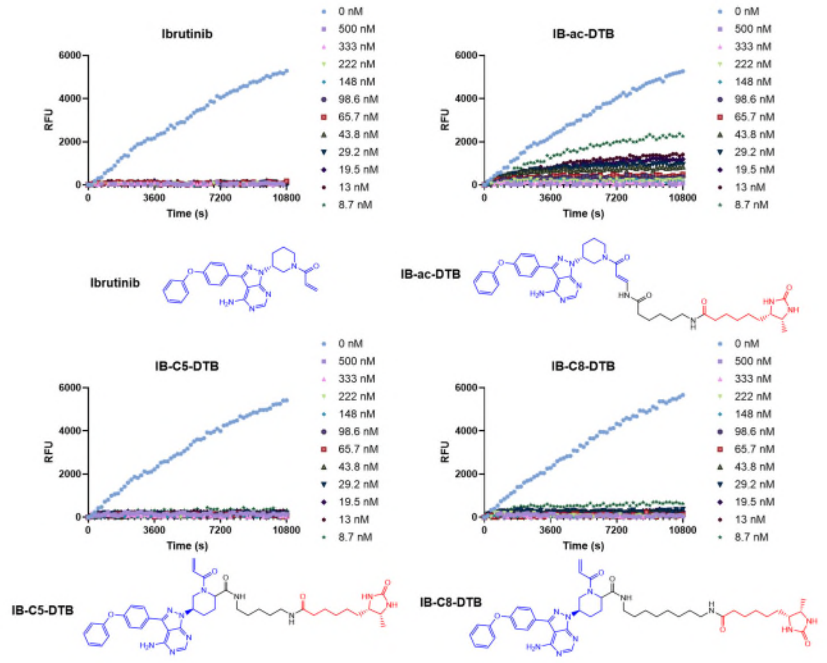

B

Reaction: 1mM Ibrutinib/IB-ac-DTB + 100mM GSH in 100mM HEPES pH 7.5/20% ACN @37 °C

	Ibrutinib		IB-ac-DTB		Blank
Incubation Time	XIC Area	LC Area	XIC Area	LC Area	GSSG/GSH %
0h	4.3e7	16318	1.2e7	8886	<1%
2h	3.7e7	13355	1.4e7	8675	5%
8h	3.7e7	11490	1.4e7	8171	20%
10h	3.2e7	11269	1.4e7	8391	40%
14h	3.3e7	11407	1.4e7	8565	66%
20h	3.5e7	11268	1.2e7	8453	100%

Supplementary Figure 2. IB-DTB probes with different BTK inhibition potency and GSH reactivity using different exit vectors.
(A) BTK kinase activity assay using PhosphoSens substrate and 1.28 nM BTK full-length protein. **(B)** GSH reactivity measured by LC-MS at intervals. IB-ac-DTB showed very weak GSH reactivity compared to ibrutinib. XIC, Extracted Ion Chromatogram.

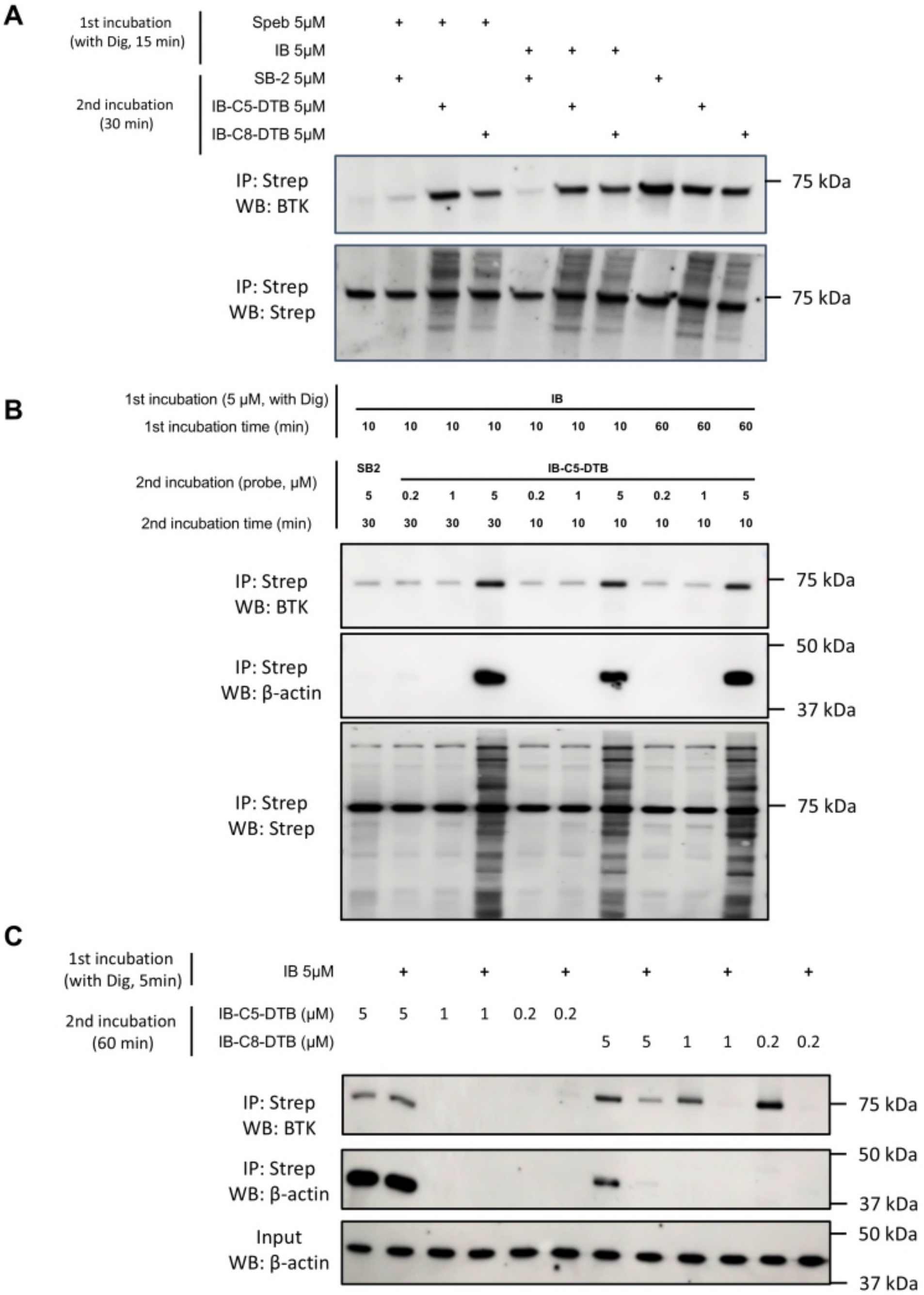

Supplementary Figure 3. Optimizing the concentration of IB-DTB probe to avoid non-specific labeling and efficient pulldown.

(A) IB-C5-DTB and IB-C8-DTB labeling at 5 μM cannot be competed out by either spebrutinib or ibrutinib. **(B)** IBC5DTB as low as 1 μM allows effective IB competition. **(C)** IB-C8-DTB, but not IB-C5-DTB, allows efficient pulldown under the native pulldown condition. IB, ibrutinib, Dig, digitonin. All the blots are representative for three independent experiments.

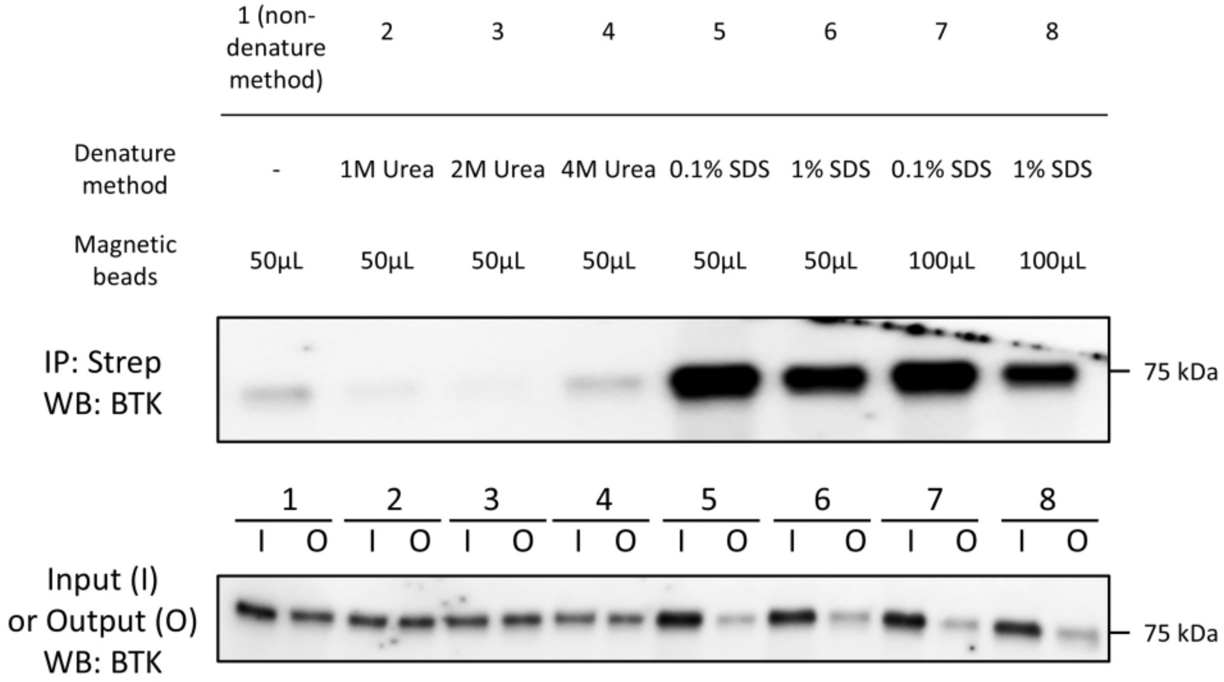

Supplementary Figure 4. Denature pulldown condition optimization for IB-C8-DTB.

A total of 2 million Ramos cells were used for each sample. Cells were lysed and incubated with 1 µM IB-C8-DTB for 1 hour at room temperature, followed by desalting and incubating overnight with indicated amount of streptavidin magnetic beads in the denature buffer containing urea or SDS. Blots are representative for two independent experiments.

A

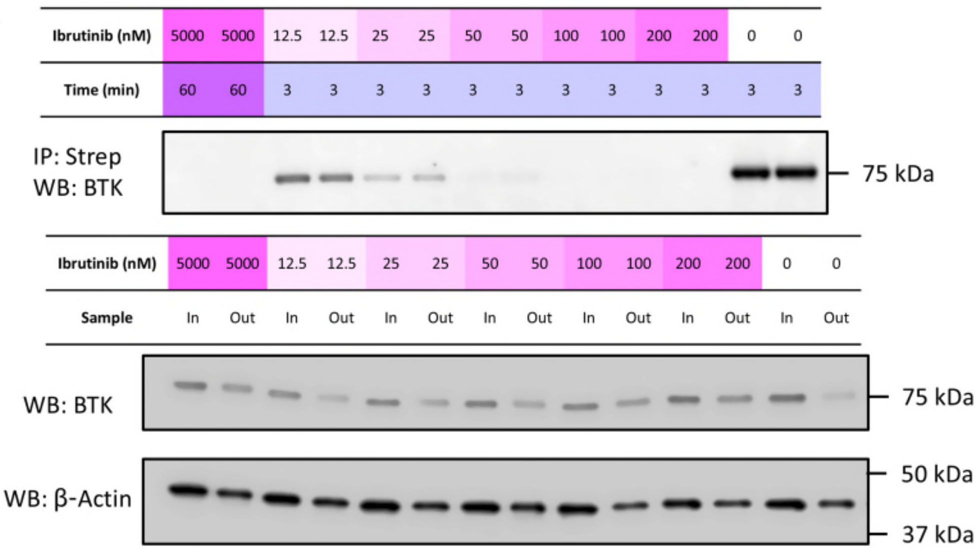

B

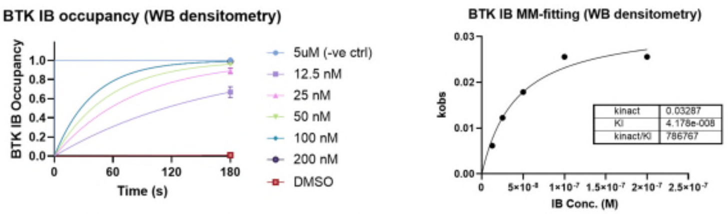

	12.5 nM	25 nM	50 nM	100 nM	200 nM
Y0	= 0.000	= 0.000	= 0.000	= 0.000	= 0.000
Plateau	= 1.000	= 1.000	= 1.000	= 1.000	= 1.000
K	0.006159	0.01226	0.01788	0.02558	0.02558

C

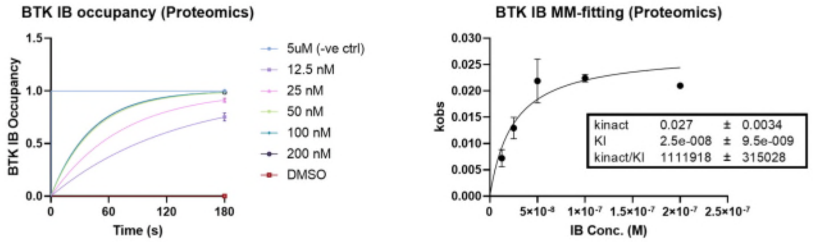

Supplementary Figure 5. Single timepoint COOKIE profiling for ibrutinib in Ramos cell using IB-C8-DTB desthiobiotin probe.

(A) Single timepoint ibrutinib COOKIE experiment layout and the immunoblots for BTK in the pulldown samples, input samples before pulldown and output samples after pulldown. Blots are representative for three independent experiments. **(B)** Densitometry analysis and **(C)** Proteomics analysis of panel A BTK pulldown sample blot plotted as first step progressive curve (left) and second step $[I] - k_{\text{obs}}$ plot fitting into Michaelis-Menton model to obtain k_{inact} and K_I values for ibrutinib against BTK. Data are presented as mean ± SD from two biological replicates.

A Zeba plate Desalting test – DTB-C6-BDP

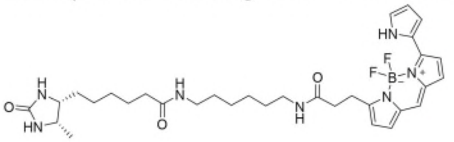

DTB-C6-BODIPY

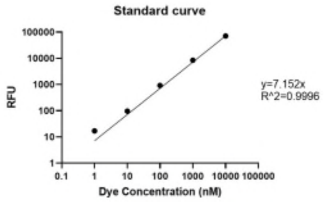

Zeba plate desalting volume µL (10 µM)	Desalted sample fluorescence (RFU)	Calculated Conc.
120	7	<1nM
160	3	<1nM
200	82	11.5nM
240	114	16nM
280	376	52nM
320	721	101nM

B

Sample ID	Digitoninized cells	Lysis buffer	Volume for Zeba plate (µL)	Streptavidin beads volume (µL)
1	10M/ml in 50µL (0.5M)	2x M-per 70µL	120	50
2	0.5M in 60µL	2x M-per 60µL	120	50
3	0.5M in 90µL	2x M-per 90µL	180	50
4	0.5M in 120µL	2x M-per 120µL	240	50
5	10M/ml in 60µL (0.6M)	2x M-per 60µL	120	50
6	10M/ml in 90µL (0.9M)	2x M-per 90µL	180	50
7	10M/ml in 120µL (1.2M)	2x M-per 120µL	240	50
8	10M/ml in 90µL (0.9M)	5x NP40 buffer 22.5µL	112.5	50
9	10M/ml in 120µL (1.2M)	5x NP40 buffer 30µL	150	50
10	10M/ml in 180µL (1.8M)	5x NP40 buffer 45µL	225	50
11	10M/ml in 60µL (0.6M)	2x M-per 60µL	120	25
12	10M/ml in 90µL (0.9M)	2x M-per 90µL	180	25
13	10M/ml in 120µL (1.2M)	2x M-per 120µL	240	25

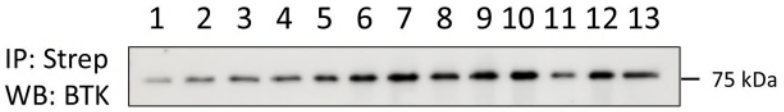

Supplementary Figure 6. Desalting condition optimization to remove DTB probes. (A). Off-label pressure test of Thermo Zeba desalting plate using increasing volume of 10 µM DTB-C6-BDP. A volume less than 200 µL can ensure 99.9% removal of small molecule dye in the sample. **(B)**. Multiple factor optimization for the pulldown protein amount reflected by the BTK WB band intensity from the IP sample. Blots are representative for two independent experiments.

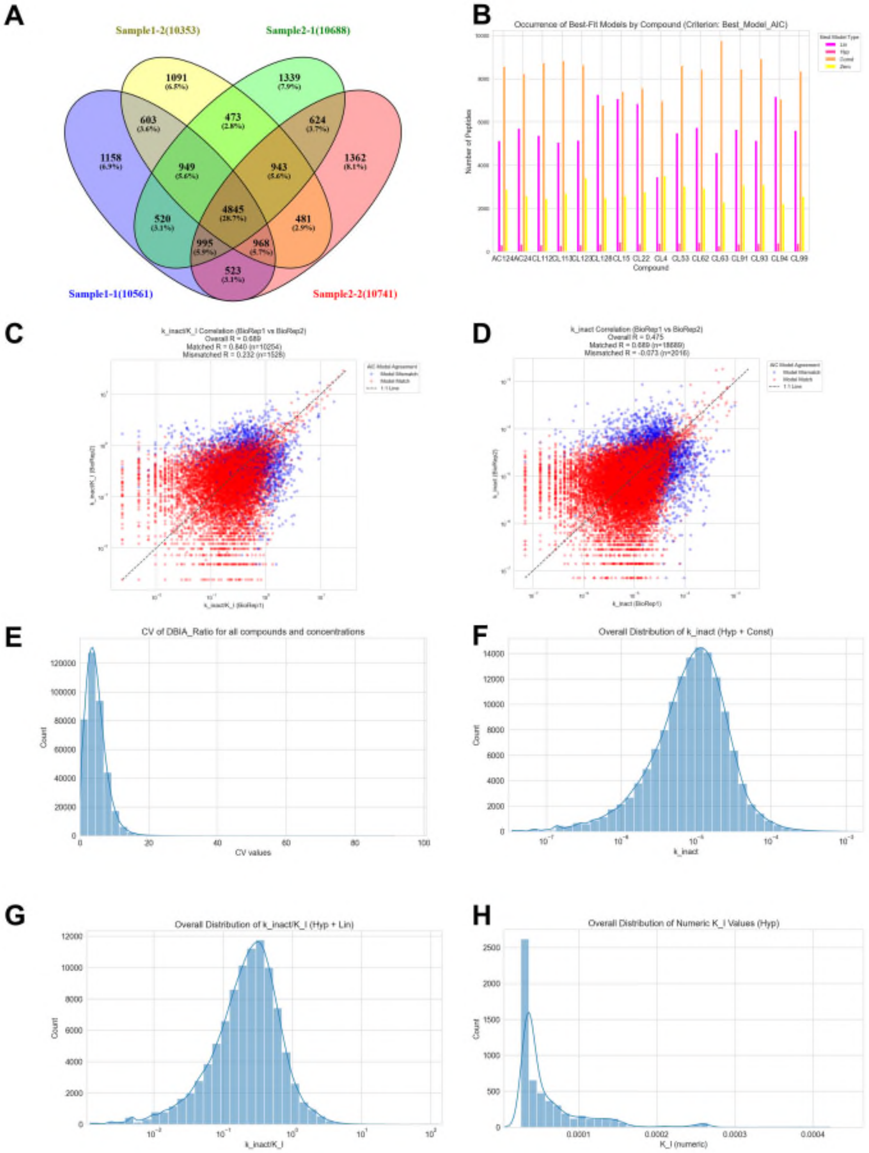

Supplementary Figure 7. Descriptive statistics for two-point COOKIE-Pro fragment screening. (A) Venn diagram illustrating the overlap of identified DBIA-labeled peptide groups across two biological replicates (sample1, sample2) and two technical replicates each (e.g., sample1-1, sample1-2). **(B)** Bar chart showing the distribution of best-fit models (Zero, Linear, Hyperbolic, Constant) for each screened fragment. **(C)** Correlation plot of calculated inactivation efficiencies ($\boldsymbol{k_{\text{inact}}/K_I}$) between two biological replicates, colored by matched/unmatched model selection for replicates. **(D)** Correlation plot of calculated reactivity ($\boldsymbol{k_{\text{inact}}}$) between two technical replicates, colored by matched/unmatched model selection for replicates. **(E)** Histogram showing the distribution of the coefficient of variation (CV) for all quantified DBIA peptide ratios across all conditions. **(F)** Histogram of the overall distribution of calculated $\boldsymbol{k_{\text{inact}}}$) values derived from peptides fitting the hyperbolic and constant models. **(G)** Histogram of the overall distribution of calculated inactivation efficiencies ($\boldsymbol{k_{\text{inact}}/K_I}$) from peptides fitting the hyperbolic and linear models. **(H)** Histogram of the overall distribution of calculated $\boldsymbol{K_I}$ values from peptides fitting the hyperbolic model.

Supplementary Methods

Chemistry

Synthesis of SB-2

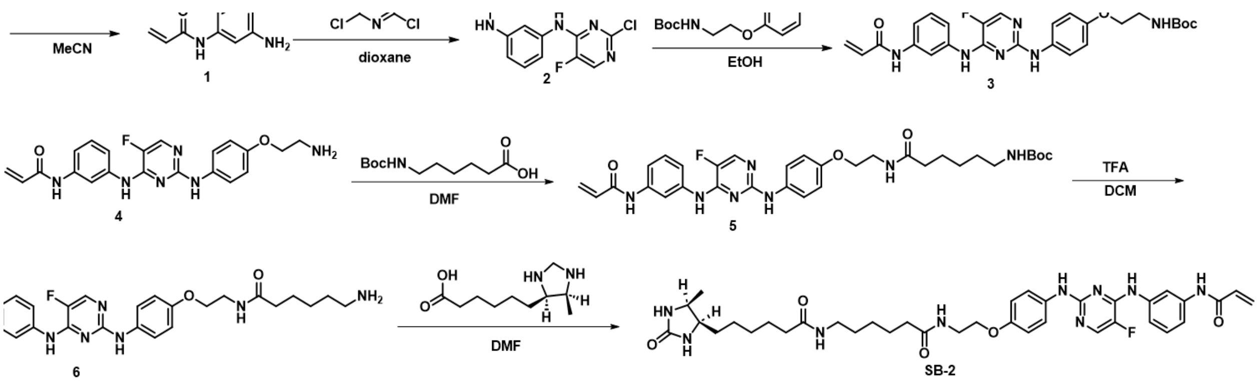

A mixture of *m*-phenylenediamine (487 mg, 4.51 mmol), acetonitrile (9 mL) and sodium bicarbonate (1.01 g, 12.00 mmol) were cooled to 0˚C in an ice bath, then acryloyl chloride (272 mg, 3.00 mmol) was added dropwise over 10 min, and the resulting mixture was allowed to warm to room temperature and stirred for another 30 min. The reaction mixture was then poured into water (60 mL), filtered and washed with water to give the crude intermediate **1**.

To a solution of 2,4-dichloropyrimidines (410 mg, 2.45 mmol) in 1,4-dioxane was added intermediate **1** (332 mg, 2.04 mmol) and DIPEA (396 mg, 3.07 mmol). The reaction mixture was stirred at 80 ˚C for 4 h. After being cooled to room temperature, the reaction mixture was added to water (30 mL). The precipitate was filtered, and the filtered cake was rinsed with additional cool water and dried in a vacuum oven to give the intermediate **2**, which were used without further purification.

To a solution of **2** (200 mg, 0.683 mmol) and *tert*-Butyl 2-(4-aminophenoxy)ethylcarbamate (276 mg, 1.09 mmol) in ethanol (4 mL) was added glacial acetic acid (20.5 mg, 0.342 mmol), and the reaction mixture was stirred in a sealed tube for 16 h at 90 ˚C. The reaction mixture was cooled, concentrated under reduced pressure. The residue was quenched with a 10% sodium bicarbonate solution (4 mL) and extracted with ethyl acetate (3x10 mL). The combined ethyl acetate was washed with water (10 mL), brine (10 mL), dried over Na_2SO_4 and concentrated under reduced pressure. The crude residue was further purified by column chromatography to get intermediate **3** (165 mg, 47%).

To a solution of **3** (165 mg, 0.324 mmol) in DCM (1 mL) was added TFA (0.5 mL). The reaction

mixture was stirred for 2 h. The mixture was concentrated under reduced pressure to get intermediate **4** (133 mg, 73 %).

To a solution of **4** (38.4 mg, 0.094 mmol) and Boc-6-aminohexanoic acid (26.1 mg, 0.113 mmol) in DMF (4 mL) was added HATU (71.5 mg, 0.188 mmol) and DIPEA (48.6 mg, 0.376 mmol). The reaction mixture was stirred at room temperature overnight. The mixture was concentrated under reduced pressure and was further purified by column chromatography to get intermediate **5** (37 mg, 63%).

To a solution of **5** (37mg, 0.324 mmol) in DCM (1 mL) was added TFA (0.5 mL). The reaction mixture was stirred for 2 h. The mixture was concentrated under reduced pressure to get intermediate **6** (26.9 mg, 97 %).

To a solution of **6** (26.9 mg, 0.052 mmol) and *d*-desthiobiotin (12.1 mg, 0.0.057 mmol) in DMF (1 mL) was added HATU (39.2 mg, 0.103 mmol) and DIPEA (33.3 mg, 0.258 mmol). The reaction mixture was stirred at room temperature overnight. The mixture was concentrated under reduced pressure and was further purified by column chromatography to get **SB-2** (31 mg, 84%).

SB2 ^{1}H NMR (400 MHz, DMSO-d_6) δ 10.22 (s, 1H), 9.37 (s, 1H), 9.00 (s, 1H), 8.07 – 8.03 (m, 2H), 7.97 (s, 1H), 7.73 (s, 1H), 7.54 (d, *J* = 9.0 Hz, 2H), 7.49 (d, *J* = 8.1Hz, 1H), 7.42 (d, *J* = 8.1 Hz, 1H), 7.27 (t, *J* = 8.1 Hz, 1H), 6.76 (d, *J* = 9.0 Hz, 2H), 6.51 – 6.45 (m, 1H), 6.30 – 6.23 (m, 2H), 6.12 (s, 1H), 5.75 (d, *J* = 9.8 Hz, 1H), 3.88 (t, *J* = 5.8 Hz, 2H), 3.61 – 3.58 (m, 2H), 2.99 (q, *J* = 6.9 Hz, 2H), 2.05 (dt, J = 23.6, 7.6 Hz, 4H), 1.47 (q, J = 7.7 Hz, 4H), 1.38 – 1.14 (mf, H), 0.94 (d, *J* = 6.5 Hz, 3H). ^{13}C NMR (100 MHz, DMSO-d_6) δ 172.39, 171.89, 163.11, 162.82, 155.67 (d, *J* = 3.0 Hz), 152.93, 149.73 (d, *J* = 10.7 Hz), 140.73 (d, *J* = 19.3 Hz), 140.33 (d, *J* = 246.0 Hz), 139.10, 139.06, 134.21, 131.93, 128.60, 126.80, 120.21, 117.25, 114.73, 114.20, 113.33, 66.51, 54.97, 50.21, 38.27, 38.20, 35.34, 35.22, 29.50, 28.97, 28.70, 26.10, 25.55, 25.22, 24.97, 15.48. HRMS (ESI) m/z: calc. for $C_{37}H_{48}FN_9O_9$ $[M + H]^+$ 718.3835, found 718.3831.

Synthesis of IB-ac-DTB

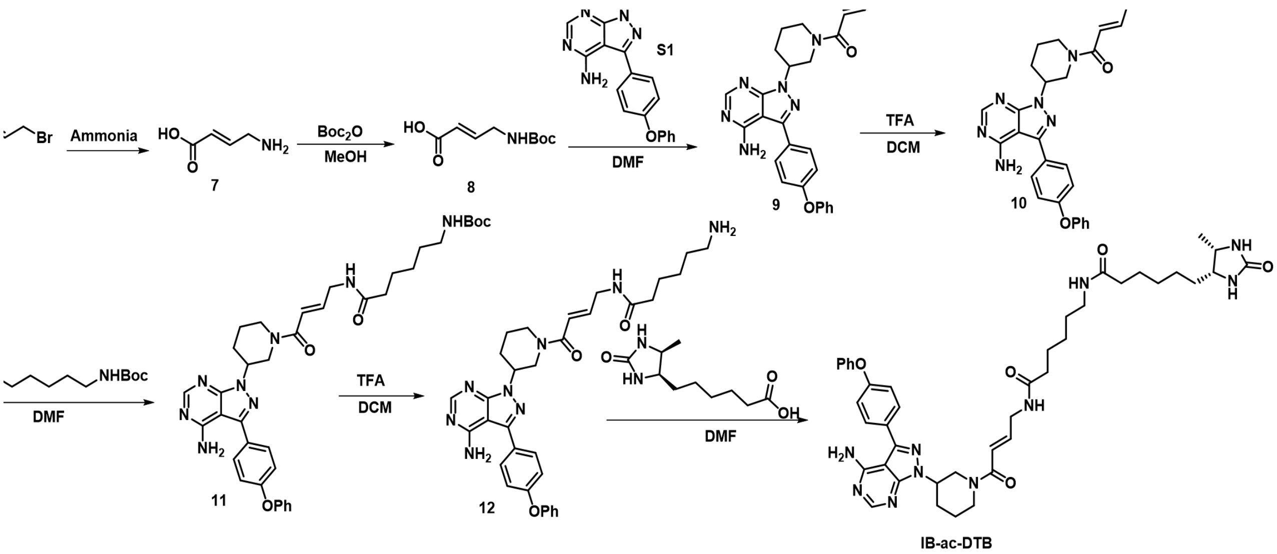

To a vial were added (E)-4-bromobut-2-enoic acid (100 mg, 0.6 mmol) and aqueous ammonia (1 mL) and was stirred at room temperature overnight. The mixture was concentrated under reduced pressure to dryness to yield the intermediate **7** (57 mg, 93%), which was used in the next step without purification.

To a vial containing a solution of **7** (57 mg, 0.56 mmol), Na_2CO_3 (120.9 mg, 1.12 mmol), THF (2 mL), and H_2O (2mL) was added Boc_2O (241.8 mg, 1.12 mmol). The reaction mixture was stirred at room temperature overnight. Then the mixture was extracted with EtOAc and the pH of the aqueous layer was adjusted to 2 with HCl (1M). The aqueous layer was extracted with EtOAc, washed with brine (3X), dried over anhydrous Na_2SO_4, filtered, and concentrated under reduced pressure to yield the intermediate **8** (96.9 mg, 86%).

To a solution of **S1** (185.2 mg, 0.48 mmol) in 10 mL DMF was added HOBT (100.7 mg,0.72 mmol), EDC (138.0 mg, 0.72 mmol), DIPEA (124.1 mg, 0.96 mmol) and **8** (96.9 mg, 0.48 mmol), successively. The reaction was stirred at room temperature overnight prior to addition of 8 mL water and then extracted with ethyl acetate (2 × 50 mL), the combined organic phase was washed with brine, dried over anhydrous Na_2SO_4 and concentrated under reduced pressure. The residue was purified by flash column to give intermediate **9** (217.7 mg, 76%).

To a solution of **9** (217.7 mg, 0.365 mmol) in DCM (5 mL) was added TFA (2 mL). The reaction

mixture was stirred for 1h. The mixture was concentrated under reduced pressure to give intermediate **10** (171.4 mg, 100%), which was used in the next step without purification.

To a solution of **10** (171.4 mg, 0.365 mmol) and Boc-6-AMinocaproic acid (101.3 mg, 0.438 mmol) in DMF (5 mL) was added HATU (277.6 mg, 0.73 mmol) and DIPEA (188.7 mg, 1.46 mmol). The reaction mixture was stirred at room temperature overnight. The mixture was concentrated under reduced pressure and was further purified by column chromatography to get intermediate **11** (107.2 mg, 42%).

To a solution of **11** (107.2 mg, 0.157 mmol) in DCM (2 mL) was added TFA (1 mL). The reaction mixture was stirred for 1 h. The mixture was concentrated under reduced pressure to give intermediate **12** (91.5 mg, 100%), which was used in the next step without purification.

To a solution of **12** (91.5 mg, 0.157 mmol) and *d*-desthiobitin (40.4 mg, 0.188 mmol) in DMF (2 mL) was added HATU (100 mg, 0.314 mmol) and DIPEA (81.2 mg, 0.628 mmol). The reaction mixture was stirred at room temperature overnight. The mixture was concentrated under reduced pressure and was further purified by column chromatography to give **IB-ac-DTB** (107.2 mg, 42%).

IB-ac-DTB ^{1}H NMR (400 MHz, Methanol-d_4) δ 8.26 (d, *J* = 11.3 Hz, 1H), 7.68 (d, *J* = 7.8 Hz, 2H), 7.40 (d, *J* = 8.0 Hz, 2H), 7.15 (d, *J* = 8.2 Hz, 3H), 7.10 (d, *J* = 8.0 Hz, 2H), 6.82 – 6.30 (m, 2H), 4.61 (s, 3H), 4.36 – 3.44 (m, 8H), 3.13 (d, *J* = 30.9 Hz, 2H), 2.63 – 1.97 (m, 8H), 1.89 – 1.20 (m, 20H), 1.09 (d, *J* = 4.6 Hz, 2H). ^{13}C NMR (100 MHz, Methanol-d_4) δ 176.08, 175.90, 167.65, 166.19, 159.96, 159.91, 157.96, 156.76, 155.16, 145.94, 142.93, 131.26, 131.10, 128.92, 125.10, 122.02, 120.54, 119.99, 99.20, 57.38, 54.36, 52.69, 51.15, 47.28, 43.60, 41.32, 40.17, 36.98, 36.80, 31.06, 30.71, 30.21, 30.04, 27.55, 27.13, 26.89, 26.56, 25.99, 24.58, 15.62. HRMS (ESI) m/z: calc. for $C_{42}H_{54}N_{10}O_5$ $[M + H]^+$ 779.4351, found 779.4342.

Synthesis of IB-C5-DTB and IB-C8-DTB

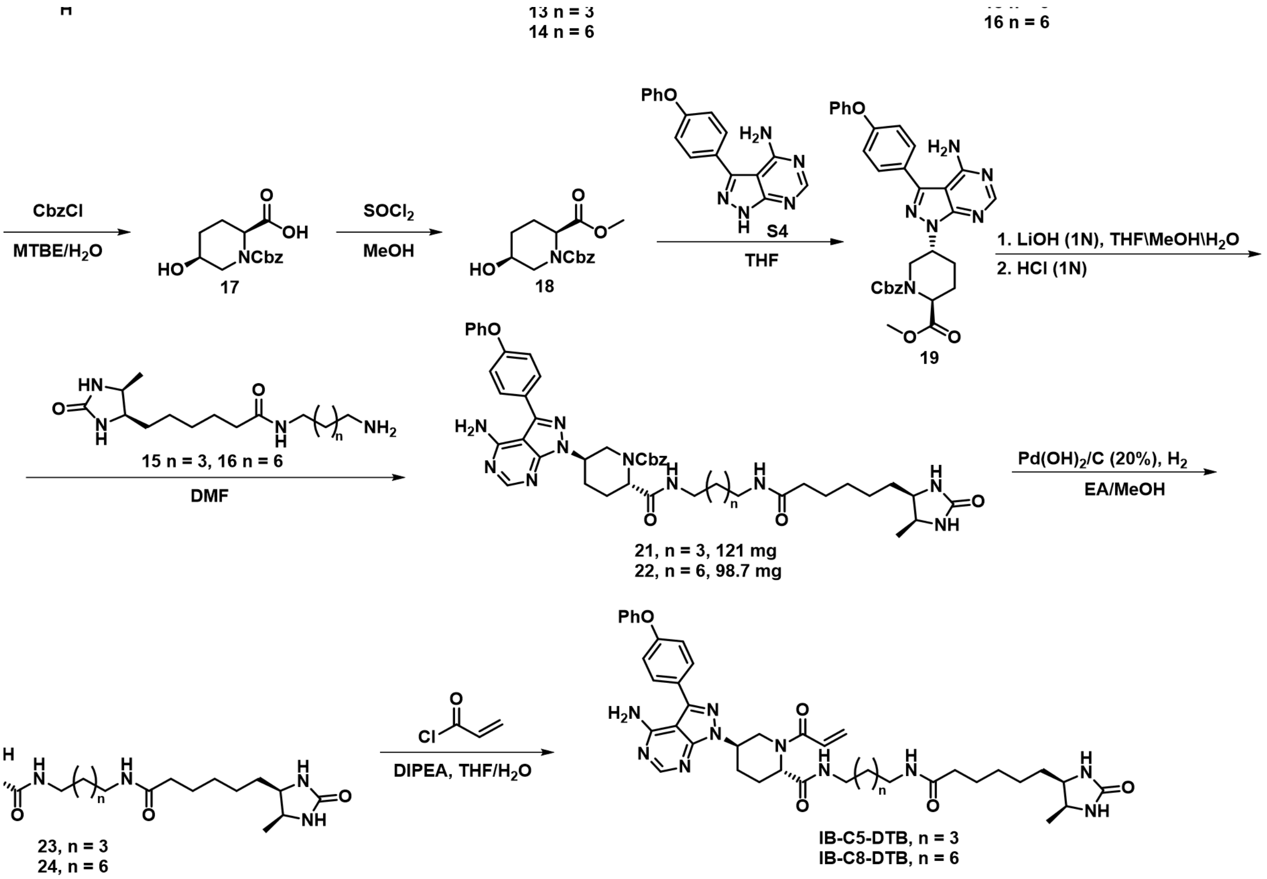

To a solution of **S2** (80.9 mg, 0.4 mmol) in DMF (2 mL) was added *d*-dethiobiotin-NHS (62.3 mg, 0.2 mmol) and DIPEA (77.5 mg, 0.6 mmol). After the solution was stirred overnight at room temperature, the solvent was removed by rotary evaporation. The residue was further purified by column chromatography to give intermediate **13** (94.7 mg, 95%).

To a solution of **S3** (97.7 mg, 0.4 mmol) in DMF (2 mL) was added *d*-dethiobiotin-NHS (62.3 mg, 0.2 mmol) and DIPEA (77.5 mg, 0.6 mmol). After the solution was stirred overnight at room temperature, the solvent was removed by rotary evaporation. The residue was further purified by column chromatography to give intermediate **14** (97.9 mg, 91%).

To a solution of **13** (94.7 mg, 0.19 mmol) in DCM (2 mL) was added TFA (1 mL). The reaction mixture was stirred for 1h. The mixture was concentrated under reduced pressure to give intermediate **15** (75.7 mg, 100%), which was used in the next step without purification.

To a solution of **14** (97.9 mg, 0.18 mmol) in DCM (2 mL) was added TFA (1 mL). The reaction

mixture was stirred for 1h. The mixture was concentrated under reduced pressure to give intermediate **15** (79.3 mg, 100%), which was used in the next step without purification.

To a solution of *cis*-5-hydroxy-L-pipecolic acid (145.2 mg, 1 mmol) and potassium carbonate (172.8 mg, 1.25 mmol) in MTBE (1 mL) and H_2O (5 mL) was added benzyl chloroformate (187.6 mg, 1.1 mmol) dropwise at 15 ˚C. After stirring for 3 h, the mixture was abstracted with MTBE (2 mL). Then the organic phase was discarded, and the aqueous phase was acidified with concentrated HCl until pH=2 at 0 ˚C. The acidified aqueous phase was extracted with ethyl acetate (3 × 10 mL), and the combined organic phase was washed with brine, dried over anhydrous Na_2SO_4 and concentrated under reduced pressure to give the crude intermediate **17**.

To a solution of **17** (145.2 mg, 1 mmol) in methanol (3 mL) was added $SOCl_2$ (130.9 mg, 1.1 mmol) dropwise under ice bath. The solution was stirred at room temperature for 4h, and then the mixture was concentrated under reduced pressure. The residue was further purified by column chromatography to give intermediate **18** (94.3 mg, 76%).

To a solution of triphenylphosphine (112.0 mg, 0.416 mmol) in THF (10 mL) was added DIAD (84.1 mg, 0.416) under ice bath. The reaction was stirred at 0 ˚C for 0.5h under N_2 atmosphere, and then a solution of **18** (94.3 mg, 0.32 mmol) in THF (4 mL) was added. The mixture was stirred at 0 ˚C for 0.5 h. After that, **S4** (211.4 mg, 0.64 mmol) was added. The reaction mixture was allowed to warm to room temperature by stirring overnight. The mixture was concentrated under reduced pressure, and the residue was purified by column chromatography to give intermediate **19** (167.8 mg, 91%).,

Intermediate **19** (167.8 mg, 0.29 mmol) was dissolved in THF (3.5 mL), MeOH (1.2 mL) and H_2O (1.2 mL). 1N LiOH (1.72 mL) was added to the reaction mixture slowly at 0 ˚C. The mixture was stirred at room temperature overnight. The mixture was acidified with HCl (1N) to pH=2 at 0 ˚C. The acidified mixture was extracted with ethyl acetate (3 × 10 mL), and the combined organic phase was washed with brine, dried over anhydrous Na_2SO_4 and concentrated under reduced pressure to give intermediate **20** (152.4 mg, 93%).

To a solution of **20** (101 mg, 0.18 mmol) and **15** (106.9 mg, 0.27 mmol) in DMF (4 mL) was added HATU (136.9 mg, 0.36 mmol) and DIPEA (116.3 mg, 0.9 mmol). The reaction mixture was stirred at

room temperature overnight. The mixture was concentrated under reduced pressure and was further purified by column chromatography to get intermediate **21** (110.2 mg, 72%).

To a solution of **20** (101 mg, 0.18 mmol) and **16** (119.0 mg, 0.27 mmol) in DMF (4 mL) was added HATU (136.9 mg, 0.36 mmol) and DIPEA (116.3 mg, 0.9 mmol). The reaction mixture was stirred at room temperature overnight. The mixture was concentrated under reduced pressure and was further purified by column chromatography to get intermediate **22** (100.7 mg, 63%).

To a solution of **21** (110.2 mg, 0.13 mmol) in methanol (2 mL) was added palladium hydroxide on activated carbon (10 mg, 10%). The mixture was stirred under H_2 atmosphere at room temperature for 3h. The mixture was filtered, and the filtrate concentrated under reduced pressure. The residue was further purified by column chromatography to get intermediate **23** (84.2 mg, 92%).

To a solution of **22** (100.7 mg, 0.11 mmol) in methanol (2 mL) was added palladium hydroxide on activated carbon (10 mg, 10%). The mixture was stirred under H_2 atmosphere at room temperature for 3h. The mixture was filtered, and the filtrate concentrated under reduced pressure. The residue was further purified by column chromatography to get intermediate **24** (97.9 mg, 100%).

To a solution of **23** (84.2 mg, 0.12 mmol) in THF (1.5 mL) was added H_2O (2 drops) and DIPEA (41.9 mg, 0.32 mmol). Acryloyl chloride (10.9 mg, 0.12 mmol) was added dropwise in an ice bath. The mixture was allowed to warm to room temperature and stirred overnight. The solution was concentrated under reduced pressure, and the residue was further purified by column chromatography to give **IB-C5-DTB** (25.6 mg, 28%).

IB-C5-DTP ^{1}H NMR (400 MHz, Methanol-d_4) δ 8.25 (s, 1H), 7.64 (d, *J* = 8.5 Hz, 2H), 7.40 (t, *J* = 7.8 Hz, 2H), 7.19 – 7.07 (m, 5H), 6.52 – 6.36 (m, 1H), 5.95 (d, *J* = 16.4 Hz, 1H), 5.46 – 5.38 (m, 1H), 5.14 (s, 2H), 4.14 (dd, *J* = 168.3, 13.1 Hz, 1H), 3.81 (m, 1H), 3.68 (q, *J* = 7.1 Hz, 1H), 3.35 (s, 3H), 3.25 (t, *J* = 6.8 Hz, 2H), 3.17 (t, *J* = 7.2 Hz, 2H), 2.30 (s, 2H), 2.19 (t, *J* = 7.5 Hz, 3H), 1.84 – 1.22 (m, 19H), 1.09 (d, *J* = 6.2 Hz, 4H). ^{13}C NMR (100 MHz, Methanol-d_4) δ 174.69, 171.68, 168.18, 164.75, 158.43, 158.39, 156.58, 155.10, 153.83, 143.99, 129.77, 129.64, 127.94, 127.66, 126.59, 123.61, 119.06, 118.48, 97.81, 55.95, 53.96, 51.26, 50.71, 46.16, 38.94, 38.78, 35.56, 29.29, 28.79, 28.67, 28.62, 25.71, 25.47, 23.98, 23.82, 21.79. HRMS (ESI) m/z: calc. for $C_{41}H_{52}N_{10}O_5$ $[M + H]^+$ 765.4195, found 765.4189.

To a solution of **24** (97.9 mg, 0.13 mmol) in THF (1.5 mL) was added H_2O (2 drops) and DIPEA (46.7 mg, 0.36 mmol). Acryloyl chloride (12.1 mg, 0.13 mmol) was added dropwise in an ice bath. The mixture was allowed to warm to room temperature and stirred overnight. The solution was concentrated under reduced pressure, and the residue was further purified by column chromatography to give **IB-C8-DTB** (51.1mg, 49%).

IB-C8-DTP ^{1}H NMR (400 MHz, Methanol-*d*$_4$) δ 8.26 (s, 1H), 7.65 (d, *J* = 8.6 Hz, 2H), 7.41 (t, *J* = 7.9 Hz, 2H) , 7.30 – 6.99 (m, 5H), 6.47 – 6.28 (m, 1H), 5.95 (d, *J* = 16.0 Hz, 1H), 5.43 (d, *J* = 8.96 Hz, 1H), 5.14 (s, 2H), 4.15 (dd, *J* = 176.5, 15.9 Hz, 2H), 3.87 – 3.75 (m, 1H), 3.75 – 3.65 (m, 2H), 3.35 (s, 5H), 3.26 – 3.13 (m, 4H), 2.30 (s, 2H), 2.18 (t, J = 7.2 Hz, 3H). 1.64 – 1.61 (m, 2H) 1.53 – 1.30 (m, 19H), 1.10 (d, *J* = 6.4 Hz, 3H). ^{13}C NMR (100 MHz, Methanol-*d*$_4$) δ 176.07, 172.98, 169.58, 166.18, 159.84, 159.79, 157.98, 156.50, 155.23, 145.39, 131.19, 131.07, 129.36, 129.06, 128.03, 125.04, 120.49, 119.90, 99.23, 68.12, 57.37, 55.80, 55.24, 52.68, 52.12, 43.77, 40.52, 40.33, 36.99, 30.74, 30.42, 30.39, 30.30, 30.21, 27.91, 27.15, 26.92, 25.41, 23.19, 15.64, 13.18. HRMS (ESI) m/z: calc. for $C_{44}H_{58}N_{10}O_5$ $[M + H]^+$ 807.4664, found 807.4663.

NMR spectra

^{1}H NMR of **SB2**

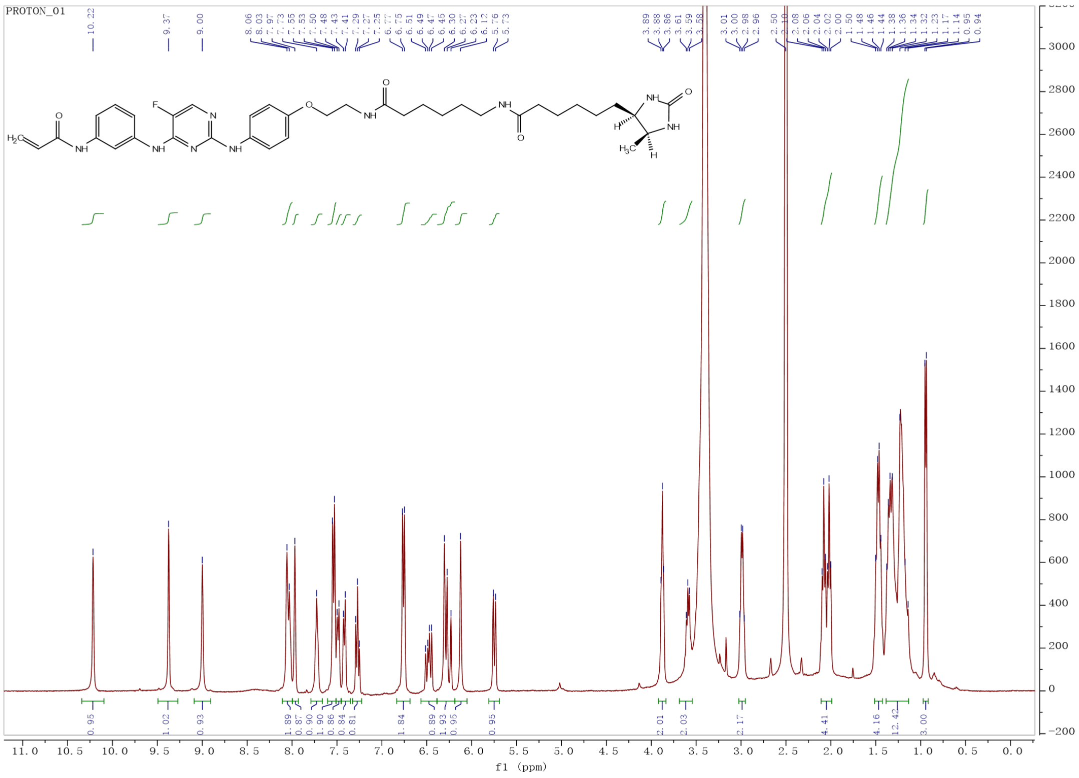

^{13}C NMR of **SB2**

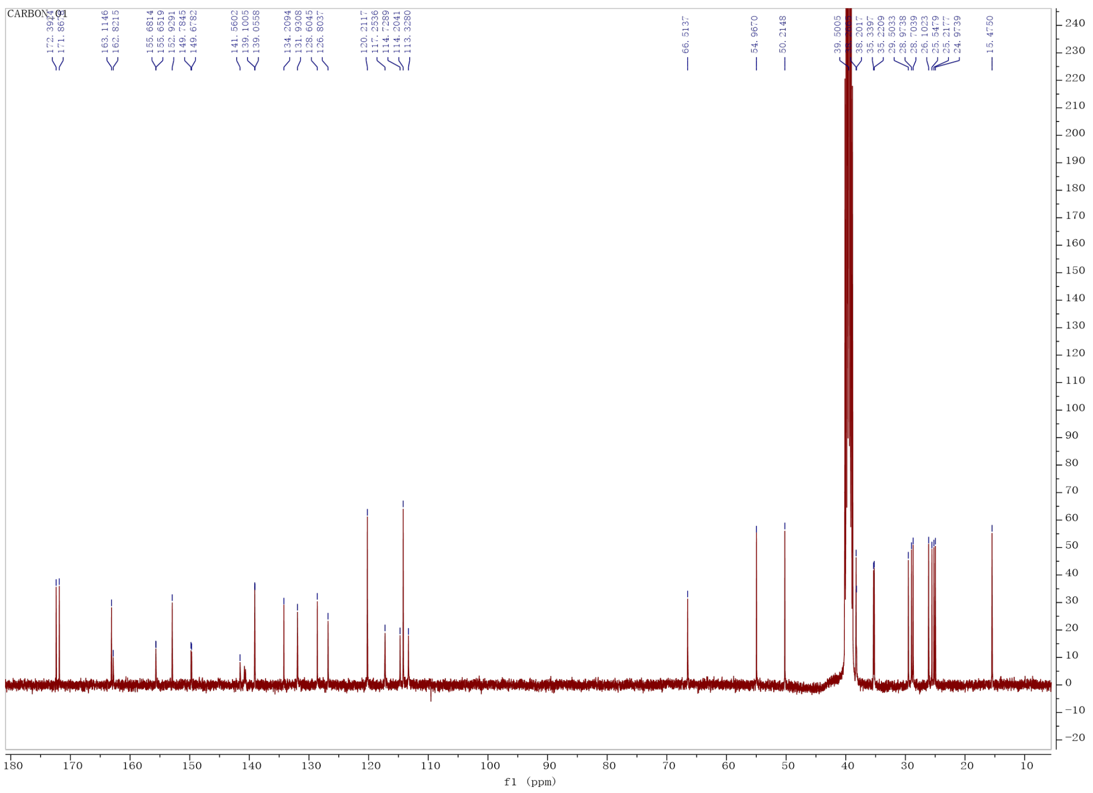

^{1}H NMR of **IB-ac-DTB**

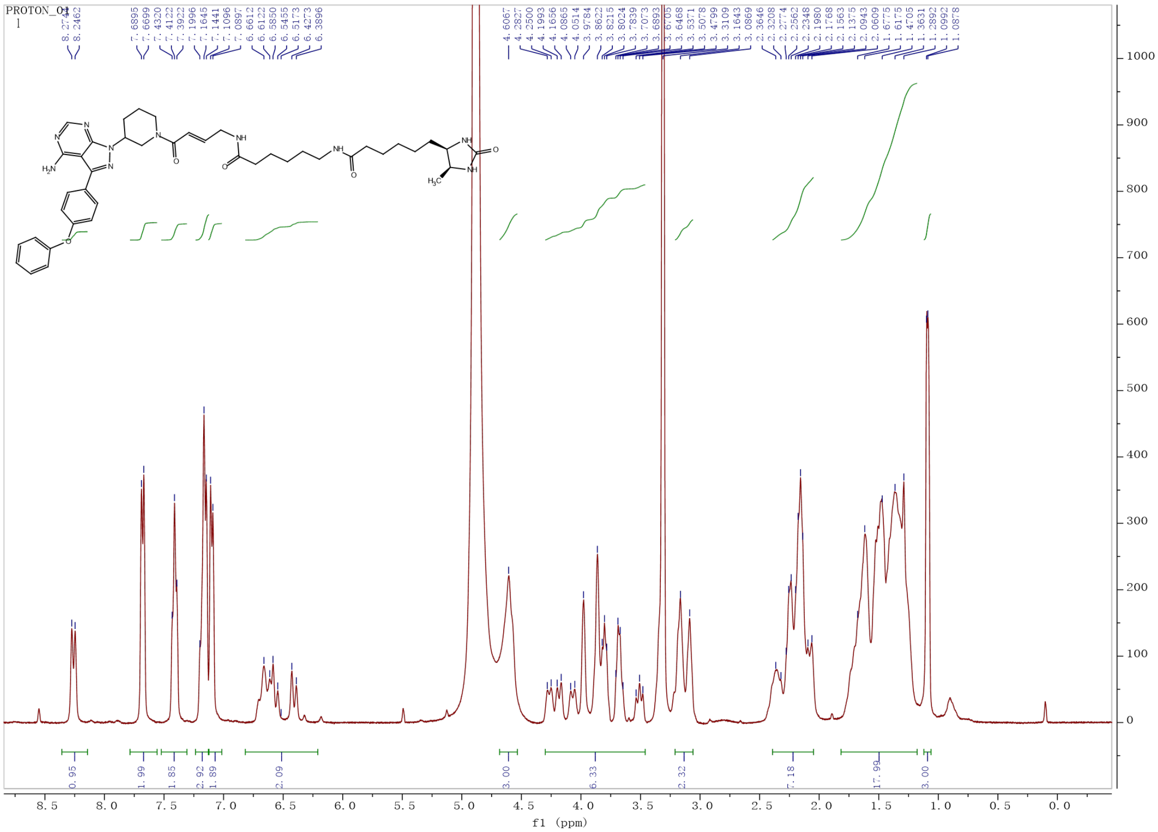

^{13}C NMR of **IB-ac-DTP**

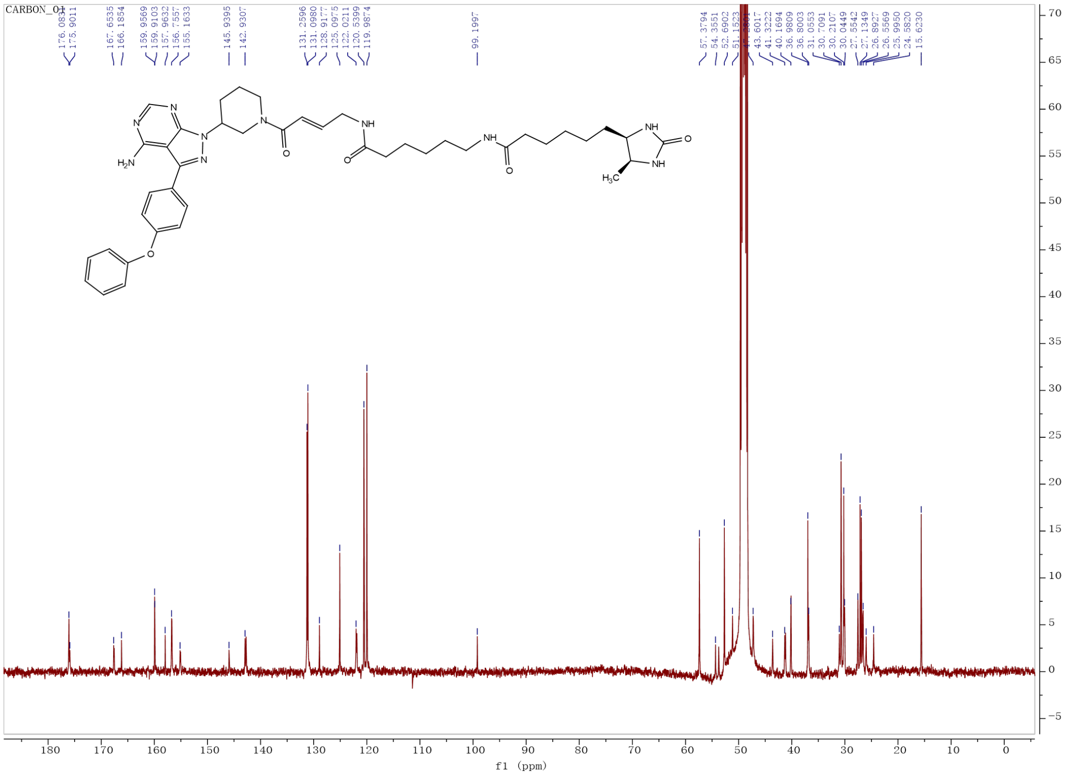

^{1}H NMR of **IB-C5-DTB**

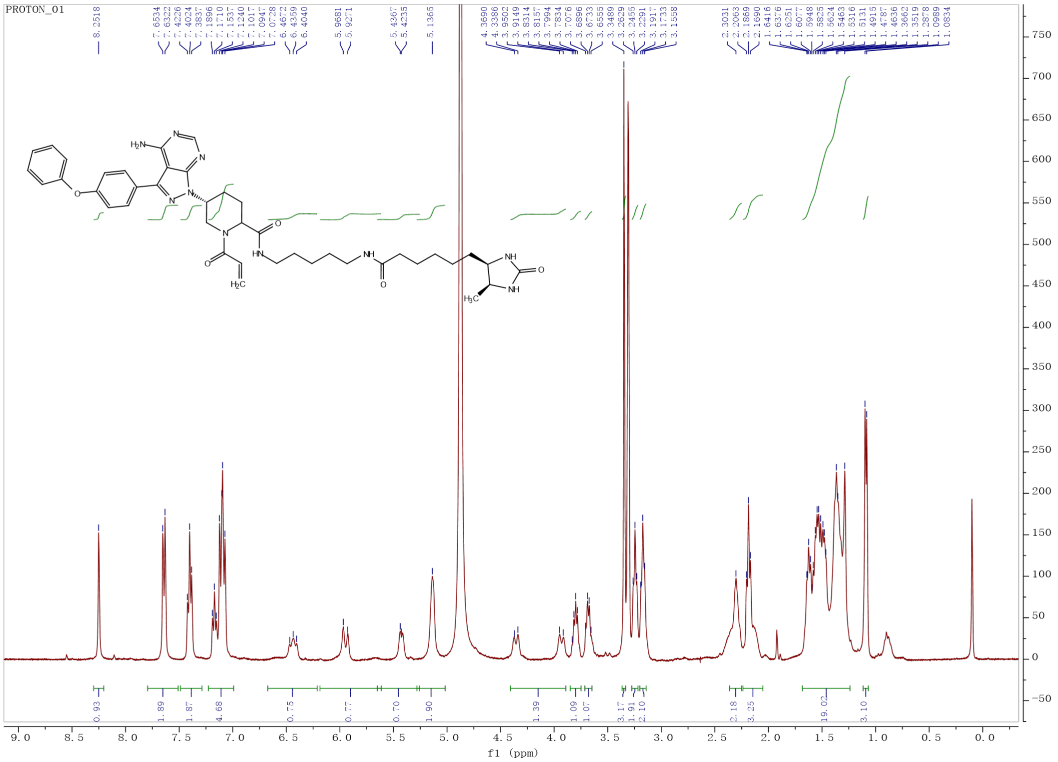

^{13}C NMR of **IB-C5-DTP**

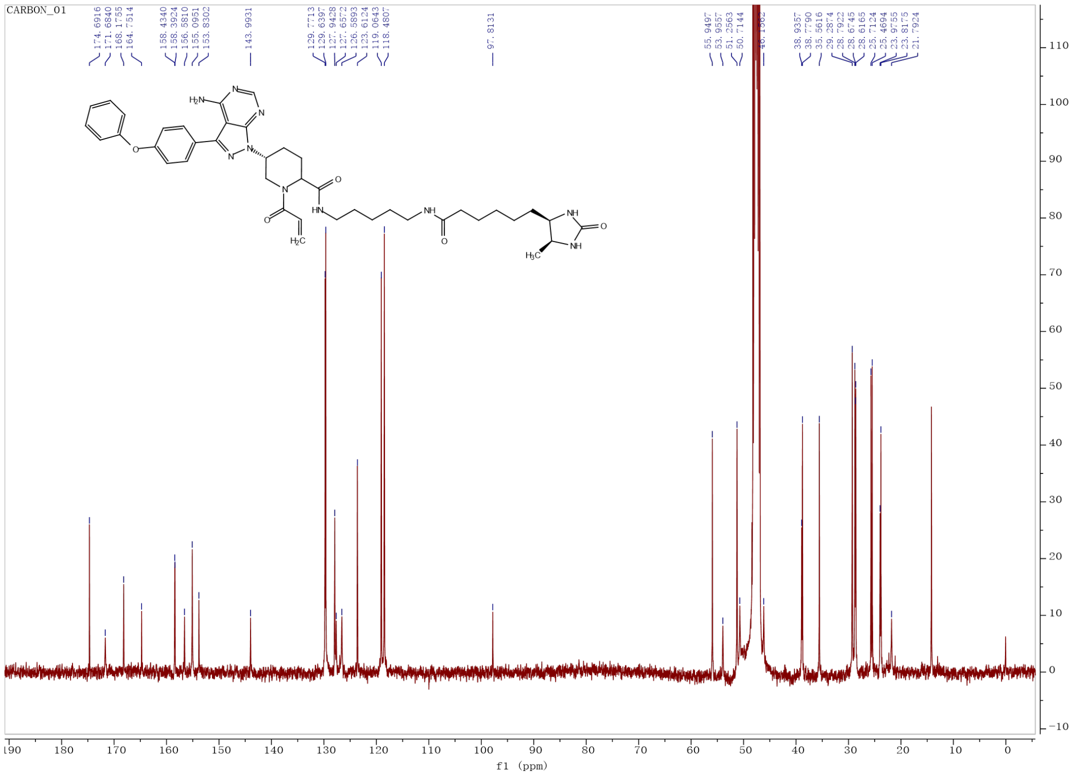

^{1}H NMR of **IB-C8-DTB**

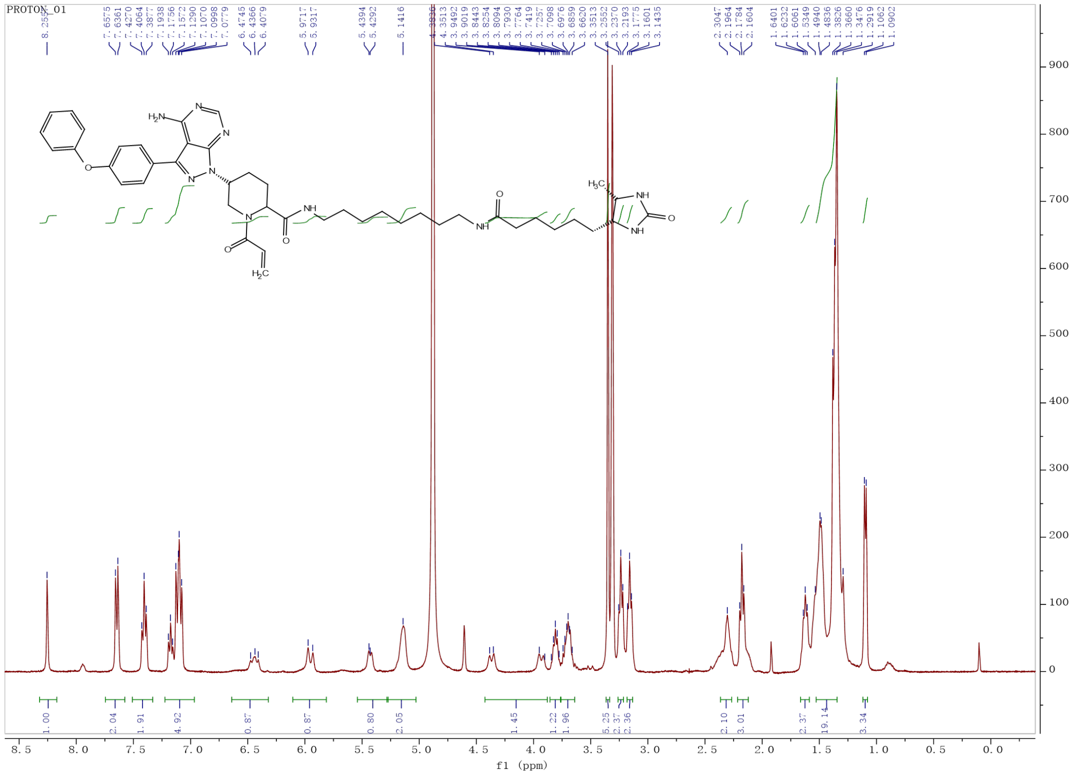

^{13}C NMR of **IB-C8-DTP**

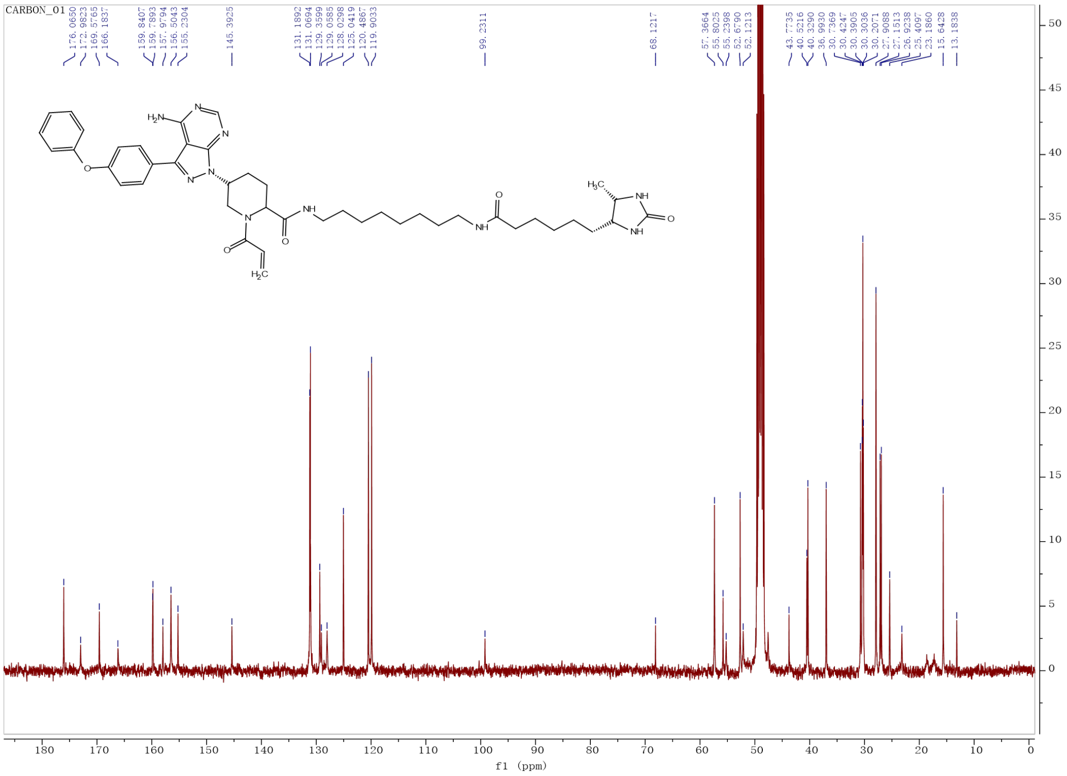

Supplement: Supplementary file 1 — Supplementary Information [file 41467_2025_63491_MOESM1_ESM.pdf]
