# Supplementary material for: COOKIE-Pro: covalent inhibitor binding kinetics profiling on the proteome scale
Source: Nat Commun. 2025 Sep 30;16:8373. doi: 10.1038/s41467-025-63491-2 (PMC12484723; doi:10.1038/s41467-025-63491-2)
Supplement: Supplementary file 2 — Description of Additional Supplementary Files [file 41467_2025_63491_MOESM2_ESM.pdf]

Description of Additional Supplementary Files

File Name: Supplementary Data 1

Description: Spebrutinib COOKIE-Pro abundance ratio double normalized with 5 uM saturation control sample and ACC1. Group description: (1) 5uM Speb 60 min (2_X) 25 nM Speb (3_X) 50 nM Speb (4_X) 100 nM Speb (5_X) 200 nM Speb (6_X) 400 nM Speb (7) DMSO control. X=1, 2 min; X=2, 4 min; X=3, 6 min.

File Name: Supplementary Data 2

Description: Ibrutinib COOKIE-Pro abundance ratio double normalized with 5 uM saturation control sample and ACC1. Group description: (1) 5uM IB 60 min (2_X) 12.5 nM IB (3_X) 25 nM IB (4_X) 50 nM IB (5_X) 100 nM IB (6_X) 200 nM IB (7) DMSO control. X=1, 2 min; X=2, 4 min; X=3, 6 min.

File Name: Supplementary Data 3

Description: KI values (M) for each cysteine-fragment pair calculated from HCT116 SLC-ABPP dataset competition ratio (CR). Only CR>2 values were used for KI conversion.

File Name: Supplementary Data 4

Description: Ligand efficiency for each cysteine-fragment pair reanalyzed from HCT116 SLC-ABPP dataset.

File Name: Supplementary Data 5

Description: Ligand lipophilic efficiency for each cysteine-fragment pair reanalyzed from HCT116 SLC-ABPP dataset.

File Name: Supplementary Data 6

Description: Two-point COOKIE-Pro kinetic params. Details available in the "Description" tab.
