# Supplementary material for: COOKIE-Pro: covalent inhibitor binding kinetics profiling on the proteome scale
Source: Nat Commun. 2025 Sep 30;16:8373. doi: 10.1038/s41467-025-63491-2 (PMC12484723; doi:10.1038/s41467-025-63491-2)
Supplement: Supplementary file 9 — Reporting Summary [file 41467_2025_63491_MOESM9_ESM.pdf]

nature portfolio

Corresponding author(s): Jin Wang

Last updated by author(s): Aug 12, 2025

Reporting Summary

Nature Portfolio wishes to improve the reproducibility of the work that we publish. This form provides structure for consistency and transparency in reporting. For further information on Nature Portfolio policies, see our Editorial Policies and the Editorial Policy Checklist.

Statistics

For all statistical analyses, confirm that the following items are present in the figure legend, table legend, main text, or Methods section.

n/a	Confirmed	
☐	☒	The exact sample size (*n*) for each experimental group/condition, given as a discrete number and unit of measurement
☐	☒	A statement on whether measurements were taken from distinct samples or whether the same sample was measured repeatedly
☒	☐	The statistical test(s) used AND whether they are one- or two-sided<br>*Only common tests should be described solely by name; describe more complex techniques in the Methods section.*
☒	☐	A description of all covariates tested
☐	☒	A description of any assumptions or corrections, such as tests of normality and adjustment for multiple comparisons
☐	☒	A full description of the statistical parameters including central tendency (e.g. means) or other basic estimates (e.g. regression coefficient) AND variation (e.g. standard deviation) or associated estimates of uncertainty (e.g. confidence intervals)
☒	☐	For null hypothesis testing, the test statistic (e.g. *F*, *t*, *r*) with confidence intervals, effect sizes, degrees of freedom and *P* value noted<br>*Give P values as exact values whenever suitable.*
☒	☐	For Bayesian analysis, information on the choice of priors and Markov chain Monte Carlo settings
☒	☐	For hierarchical and complex designs, identification of the appropriate level for tests and full reporting of outcomes
☒	☐	Estimates of effect sizes (e.g. Cohen's *d*, Pearson's *r*), indicating how they were calculated

Our web collection on statistics for biologists contains articles on many of the points above.

Software and code

Policy information about availability of computer code

Data collection	Mass spectra were obtained using an Orbitrap Fusion Lumos and Orbitrap Ascend mass spectrometer (Thermo Fisher Scientific)
Data analysis	Raw spectra were analyzed using Proteome Discoverer 2.4. Two-point COOKIE-Pro was analyzed by Proteome Discoverer 3.2. COOKIE-Pro data analysis protocol has been detailedly explained in the method part and the scripts used has been deposited onto GitHub https://github.com/Hanfeng-Lin/COOKIE_scripts. MATLAB (version R2022b) and the SimBiology package (v6.4) were used to build and simulate the covalent inhibition model as shown in Eq. 1. The reported standard error of k_inact and K_I was calculated by GraphPad 10.2 using symmetrical approximate confidence intervals. Reported protein abundance for each TMT channel was processed for COOKIE two-step fitting in Prism v10.2 (GraphPad Software).

For manuscripts utilizing custom algorithms or software that are central to the research but not yet described in published literature, software must be made available to editors and reviewers. We strongly encourage code deposition in a community repository (e.g. GitHub). See the Nature Portfolio guidelines for submitting code & software for further information.

Data

Policy information about availability of data

All manuscripts must include a data availability statement. This statement should provide the following information, where applicable:

- Accession codes, unique identifiers, or web links for publicly available datasets
- A description of any restrictions on data availability
- For clinical datasets or third party data, please ensure that the statement adheres to our policy

The mass spectrometry raw files for COOKIE-Pro quantitative multiplexed proteomics have been deposited in the MassIVE dataset under accession number MSV000095804 [https://massive.ucsd.edu/ProteoSAFe/dataset.jsp?accession=MSV000095804]. The processed proteomics datasets are available in Supplementary TableData S1, S2 and S6. The reanalysis result of SLC-ABPP data are available in Supplementary TableData S3-S5. Source data are provided with this paper.

Research involving human participants, their data, or biological material

Policy information about studies with human participants or human data. See also policy information about sex, gender (identity/presentation), and sexual orientation and race, ethnicity and racism.

Reporting on sex and gender	N/A
Reporting on race, ethnicity, or other socially relevant groupings	N/A
Population characteristics	N/A
Recruitment	N/A
Ethics oversight	N/A

Note that full information on the approval of the study protocol must also be provided in the manuscript.

Field-specific reporting

Please select the one below that is the best fit for your research. If you are not sure, read the appropriate sections before making your selection.

☒ Life sciences ☐ Behavioural & social sciences ☐ Ecological, evolutionary & environmental sciences

For a reference copy of the document with all sections, see nature.com/documents/nr-reporting-summary-flat.pdf

Life sciences study design

All studies must disclose on these points even when the disclosure is negative.

Sample size	No statistical method was used to determine sample size. Due to the limited available TMT quantification channels, each dose and time dependent incubation was performed in single, but multiple biological replicated were performed to ensure reproducibility and allow statistical analysis.
Data exclusions	No data exclusion was performed.
Replication	COOKIE-Pro result for spebrutinib was reproduced in all three independent experiments. COOKIE-Pro result for ibrutinib was reproduced in all two independent experiments. Two-point COOKIE-Pro fragment screening was performed in two biological replicates, and each sample injected twice for LC-MS/MS analysis.
Randomization	No randomization needed for this study. The sample was homogeneous with respect to possible confounders (same cell line). For data analysis, batch effect was treated as a covariate and adjusted for using Acetyl-CoA carboxylase 1, an endogenously biotinylated protein for normalization across TMT sets. This approach ensured that differences in protein enrichment were attributable to treatment rather than variability in sample preparation or mass spectrometer performance.
Blinding	Investigators were not blinded to sample allocation in TMT18pro channel assignment. All channels are reacted with a saturating amount of TMT reagent to ensure complete labeling of peptide amines. The mass spectrometer records and processes spectra without operator interpretation, and downstream data analysis was conducted using predefined scripts and statistical pipelines that do not require subjective judgment. As a result, there was no opportunity for observer bias to influence the experimental outcomes.

Reporting for specific materials, systems and methods

We require information from authors about some types of materials, experimental systems and methods used in many studies. Here, indicate whether each material, system or method listed is relevant to your study. If you are not sure if a list item applies to your research, read the appropriate section before selecting a response.

Materials & experimental systems

n/a	Involved in the study	
	x	Antibodies
	x	Eukaryotic cell lines
x		Palaeontology and archaeology
x		Animals and other organisms
x		Clinical data
x		Dual use research of concern
x		Plants

Methods

n/a	Involved in the study	
x		ChIP-seq
x		Flow cytometry
x		MRI-based neuroimaging

Antibodies

Antibodies used	1:1000 dilution of Cell Signaling Technology BTK Rabbit mAb#8547 (Clone:D3H5, Lot:18), 1:2000 dilution of β-actin Rabbit mAb#4970 (Clone:13E5, Lot:19); 1:1000 dilution of Abclonal: Tec Rabbit mAb A22074 (Clone:ARC53949), Lot 3522071376
Validation	Validation of each antibody was performed by the vendor Cell Signaling Technologies using their Hallmarks of Antibody Validation™ (https://www.cellsignal.com/products/8547/datasheet?images=1&protocol=0 ; https://www.cellsignal.com/products/4970/datasheet?images=1&protocol=0) or on the Abclonal website (https://static.abclonal.com/datasheet/A22074.pdf). All these antibody Western Blot trend for COOKIE samples aligned well with TMT proteomics data, indicating the specificity of these antibodies.

Eukaryotic cell lines

Policy information about cell lines and Sex and Gender in Research

Cell line source(s)	Ramos and Hela cell line was purchased from American Type Culture Collection (ATCC, Cat. No. CRL-1596 and CCL-2).
Authentication	Cells were authenticated at their source (ATCC). No extra authentication was employed in this study.
Mycoplasma contamination	Routine mycoplasma contamination test results are negative before and after all the cell-related experiments.
Commonly misidentified lines (See ICLAC register)	No commonly misidentified lines were used.

Plants

Seed stocks	N/A
Novel plant genotypes	N/A
Authentication	N/A
