# Supplementary material for: COOKIE-Pro: covalent inhibitor binding kinetics profiling on the proteome scale
Source: Nat Commun. 2025 Sep 30;16:8373. doi: 10.1038/s41467-025-63491-2 (PMC12484723; doi:10.1038/s41467-025-63491-2)
Supplement: Supplementary file 10 — Transparent Peer Review file [file 41467_2025_63491_MOESM10_ESM.pdf]

nature portfolio

Peer Review File

COOKIE-Pro: Covalent Inhibitor Binding Kinetics Profiling on the Proteome Scale

Corresponding Author: Professor Jin Wang

This file contains all reviewer reports in order by version, followed by all author rebuttals in order by version.

Version 0:

Reviewer comments:

Reviewer #1

(Remarks to the Author)
The authors report a new method for irreversible covalent ligand binding kinetics measurements. The underlying principles are clearly explained and the validations seem solid. However, I think the subject reported in the paper lacks broader interest for consideration by Nature Communications. I recommend publication in a more domain specific journal. In addition, a few comments about the scientific contents which the authors are encouraged to improve in the revisions:

(1) There are two types of covalent ligands, reversible covalent ligands and irreversible covalent ligands. The paper is only dealing with irreversible covalent ligands, but the presentations in the paper make it sound like it is for covalent ligand in general.

(2) A short review about other methods available for covalent binding kinetics measurement and comparisons with existing method would be useful for the readers to better understand what is new in the method, and when/why we should use the new method.

(3) While the validation on BTK ligands seem solid, the extended validation on the "proteome scale" as indicated in the title is very weak. I was expecting reports of kinetics measurements for some standard covalent warhead library on the proteome scale, and key findings on some interesting warheads or proteins. In the current form, the paper just reports a new method that has the potential to be applied at scale, but its usefulness remains to be validated.

Reviewer #2

(Remarks to the Author)
In this manuscript, Lin et al. developed the LC-MS-based COOKIE-Pro method which enables the determination of kinetic parameters of covalent inhibitors binding to proteins both on-target and off-targets in a proteome-wide fashion. The authors also argued that the model is broadly applicable for reactive cysteine profiling datasets by correlating the physical properties of the structures to their kinetic parameters. The manuscript is written in a very didactic manner making it easy to follow and understand. The authors showcase their method using the BTK inhibitors spebrutinib and ibrutinib and obtained on-par kinact/KI values with the literature.

While it was straightforward with spebrutinib, for ibrutinib the authors had to design and test serval probes to identify the one that would fit the requirement to accurately measure kinact/KI for the COOKIE-Pro method to work, making it difficult a one-fits-all approach with a generic probe like IAA. This example illustrates how important probe design is but presents a limitation of this method. While ibrutinib is less selective than spebrutinib, it is still a highly optimized drug molecule and therefore this raises doubt on the usage of COOKIE-Pro for compounds that are more promiscuous such as hit fragments from screening campaigns or natural product-based scaffolds. An example like that would greatly strengthen the manuscript.

Another drawback is the need of many different concentrations and time points. While the authors end the manuscript explaining this limitation and how to address it in theory (Figure 6), a real-life example would probably be a mix of these scenarios, different for each target. The number of technical replicates in this study is ambiguous. The authors should comment on how many replicates were performed for each data point and calculate CVs to demonstrate the reproducibility of the calculated kinetic parameters.

Finally, as outlined at the beginning, the probe concentration used should saturate the targets but it is not possible to know when this is achieved unless the probe binds as potently as the unmodified inhibitor. This makes it impossible to use the COOKIE-Pro method as the first line of screening and requires extensive synthesis experience and time investment to measure kinact/KI compared to more traditional methods.

In light of all these uncertainties and limitations, I believe that this work is too premature to be published in Nat Commun.

Reviewer #3

(Remarks to the Author)
I co-reviewed this manuscript with one of the reviewers who provided the listed reports. This is part of the Nature Communications initiative to facilitate training in peer review and to provide appropriate recognition for Early Career Researchers who co-review manuscripts.

Reviewer #4

(Remarks to the Author)
Noteworthy Results:
- The development of COOKIE-Pro represents an advance in profiling covalent inhibitor binding kinetics across the proteome, addressing a key challenge in the field. I can see this technique being used to normalize competition ratios based on the intrinsic reactivity of the electrophile.
- The method successfully determined kinact and KI values for both intended targets and off-targets of BTK inhibitors
- The suggested compatibility with SLC-ABPP datasets substantially enhances the broader utility of this approach.

Methodology Soundness:
The experimental approach appears to be generally robust, with appropriate controls and validation. Some suggestions for improvement:
- Test the method with a broader range of covalent inhibitors beyond BTK inhibitors. In particular, the authors should validate one or more inhibitors found from SLC-ABPP, or equivalent proteome-wide screens to back up their assumptions about shared K_inact and that COOKIE-PRO is compatible with single-dose, single-timepoint SLC-ABPP. Most covalent chemoproteomics screens are not identifying compounds as selective or potent as the clinical compounds used in this study. While the authors suggest that the method is compatible with SLC-ABPP, without experimental validation there is no evidence to support this.
- Include error analysis for the two-point method to better understand its limitations. As above, the authors should validate a protein-ligand interaction from screening using the two-point method.

Data Analysis:
- Source code must be made available. Preferably, the code could be converted to Python or R, so that the analysis pipeline here could have the broadest impact.
- Line 722 states "high peptide confidence". What is the FDR cutoff (e.g. q-value <= 0.01), and at what level is the cutoff applied (e.g. PSM-level, peptide-level, protein-level)?
- Line 725 of the manuscript states "Protein quantification uses both unique and random peptides" - I can only assume that 'random' is a typo.
- Include statistical analysis of reproducibility across biological replicates. Figs 3 and 4 should show biological replicates/error-bars.

Conclusion:
The work presented here is of good quality. Some proof-reading is necessary (typos, grammatical errors, such as on line 230). However, the addition of the SLC-ABPP compability & two-point screening seems almost like an afterthought given the absence of experimental data supporting the suggested compability. Given that compability with proteome-wide screening methods like SLC-ABPP would substantially enhance the utility of COOKIE-PRO, I think this should be addressed if the manuscript is to be published in Nature Communications. Without validation, this manuscript is essentially a technical note. Additionally, the absence of source code necessary to reproduce the analysis should prevent publication, given the central role of the analysis in this work.

Version 1:

Reviewer comments:

Reviewer #1

(Remarks to the Author)
The revised manuscript has addressed my comments. I have no other comments.

(Remarks on code availability)

Reviewer #2

(Remarks to the Author)
The authors have thoroughly addressed all of my concerns in the revised manuscript. The addition of the fragment screening experiment, the detailed analysis of reproducibility, and the clarification of probe design requirements significantly strengthen the work. I now believe the data is robust, and the methodology is sufficiently validated for broad applicability. In my view, the manuscript is mature and suitable for publication in Nature Communications.

(Remarks on code availability)

Reviewer #3

(Remarks to the Author)
I co-reviewed this manuscript with one of the reviewers who provided the listed reports. This is part of the Nature Communications initiative to facilitate training in peer review and to provide appropriate recognition for Early Career Researchers who co-review manuscripts.

(Remarks on code availability)

Reviewer #4

(Remarks to the Author)
Noteworthy Results:
- The development of COOKIE-Pro represents an advance in profiling covalent inhibitor binding kinetics across the proteome, addressing a key challenge in the field. I can see this technique being used to normalize competition ratios based on the intrinsic reactivity of the electrophile.
- The method successfully determined kinact and KI values for both intended targets and off-targets of BTK inhibitors
- The suggested compatibility with SLC-ABPP datasets substantially enhances the broader utility of this approach.

Methodology Soundness:
The experimental approach appears to be generally robust, with appropriate controls and validation. Some suggestions for improvement:
- The Authors have added in a more in-depth analysis of the method applied proteome-wide, highlighting a case study with MGMT_C145. Instead of just listing literature values, the Authors should experimentally validate MGMT_C145 kinetic parameters as they did for TEC/BTK/BLK. A full dose/time response curve that produces similar results for to the two-point screening method would highlight the reliability of the approach and, in my opinion, substantially improve the paper (e.g. Fig 4D).

(Remarks on code availability)
Thank you for providing code. It appears to be well-annotated and clear. It would be great to have the versions of the dependencies pinned in the README file or in a separates requirements.txt file.

Open Access This Peer Review File is licensed under a Creative Commons Attribution 4.0 International License, which permits use, sharing, adaptation, distribution and reproduction in any medium or format, as long as you give appropriate credit to the original author(s) and the source, provide a link to the Creative Commons license, and indicate if changes were made.
In cases where reviewers are anonymous, credit should be given to 'Anonymous Referee' and the source.
The images or other third party material in this Peer Review File are included in the article's Creative Commons license, unless indicated otherwise in a credit line to the material. If material is not included in the article's Creative Commons license and your intended use is not permitted by statutory regulation or exceeds the permitted use, you will need to obtain permission directly from the copyright holder.
To view a copy of this license, visit https://creativecommons.org/licenses/by/4.0/

REVIEWER COMMENTS

Reviewer #1 (Remarks to the Author):

The authors report a new method for irreversible covalent ligand binding kinetics measurements. The underlying principles are clearly explained and the validations seem solid. However, I think the subject reported in the paper lacks broader interest for consideration by Nature Communications. I recommend publication in a more domain specific journal. In addition, a few comments about the scientific contents which the authors are encouraged to improve in the revisions:

(1) There are two types of covalent ligands, reversible covalent ligands and irreversible covalent ligands. The paper is only dealing with irreversible covalent ligands, but the presentations in the paper make it sound like it is for covalent ligand in general.

Thank you for pointing out. We added the following statement in the abstract and the last paragraph of introduction to clarify that the COOKIE-Pro presented in the current research only works for irreversible covalent inhibitors:

Here, we report COOKIE-Pro (COvalent Occupancy KInetic Enrichment via Proteomics), an unbiased method for irreversible covalent inhibitor binding kinetics profiling on the proteome scale.

----

(2) A short review about other methods available for covalent binding kinetics measurement and comparisons with existing method would be useful for the readers to better understand what is new in the method, and when/why we should use the new method.

We have two paragraphs in the introduction part reviewing the current methods determining covalent binding kinetic parameters for enzyme target (co-incubation and pre-incubation method) and non-enzyme target protein (chemical probe-based competition assays, and MS-based method). The pros and cons were also discussed in place.

We added the following statement as a comparison of COOKIE-Pro method with the current methods:

Here, we report COOKIE-Pro (COvalent Occupancy KInetic Enrichment via Proteomics), an unbiased method for irreversible covalent inhibitor binding kinetics profiling on the proteome scale, which is suitable for both enzyme and non-enzyme targets, and eliminating the need for individual protein purification by enabling direct treatment of cell lysate.

----

(3) While the validation on BTK ligands seem solid, the extended validation on the "proteome scale" as indicated in the title is very weak. I was expecting reports of kinetics measurements for some standard covalent warhead library on the proteome scale, and key findings on some interesting warheads or proteins. In the current form, the paper just reports a new method that has the potential to be applied at scale, but its usefulness remains to be validated.

We thank the reviewer for this insightful comment. We agree that the initial manuscript, while thoroughly validating the COOKIE-Pro methodology with the BTK inhibitors spebrutinib and ibrutinib, did not sufficiently demonstrate the "proteome scale" application as suggested by the title. The reviewer's expectation to see kinetic measurements for a broader library of covalent fragments to truly validate the method's usefulness is well-taken.

To address this weakness, we have significantly expanded the manuscript to include a new section demonstrating a high-throughput application of our method. We developed a "two-point COOKIE-Pro" strategy designed for screening purposes and applied it to a library of 16 covalent fragments in HeLa cell lysate. This new experiment was designed specifically to validate the utility of our approach at the proteome scale.

The results of this new study, presented in the new Figure 7, showcase the power of this approach. From this single, high-throughput experiment, we generated approximately 128,000 potential kinetic curves and successfully fitted over 5,600 Michaelis-Menton curves across the 16 fragments. This allowed us to generate a broad landscape of kinetic parameters, including both the inactivation rate (kinact) and the inactivation efficiency (kinact/KI).

This large-scale analysis revealed key findings, as the reviewer had hoped. For instance, we identified several proteins in the thioredoxin domain-containing (TXNDC) family as frequent "hotspots" for multiple fragments. Our kinetic analysis provided the mechanistic insight that the high potency against these sites was primarily driven by the high intrinsic reactivity of the cysteine residues (a high kinact) rather than strong, specific non-covalent binding (a

low KI). This ability to decouple reactivity from affinity at a proteome-wide scale is a key advantage of our method and provides crucial information for medicinal chemists to prioritize hits for further development.

We believe these substantial additions to the manuscript now robustly demonstrate that COOKIE-Pro is not just a method with *potential*, but a validated and useful platform for profiling covalent inhibitor kinetics on a proteome-wide scale.

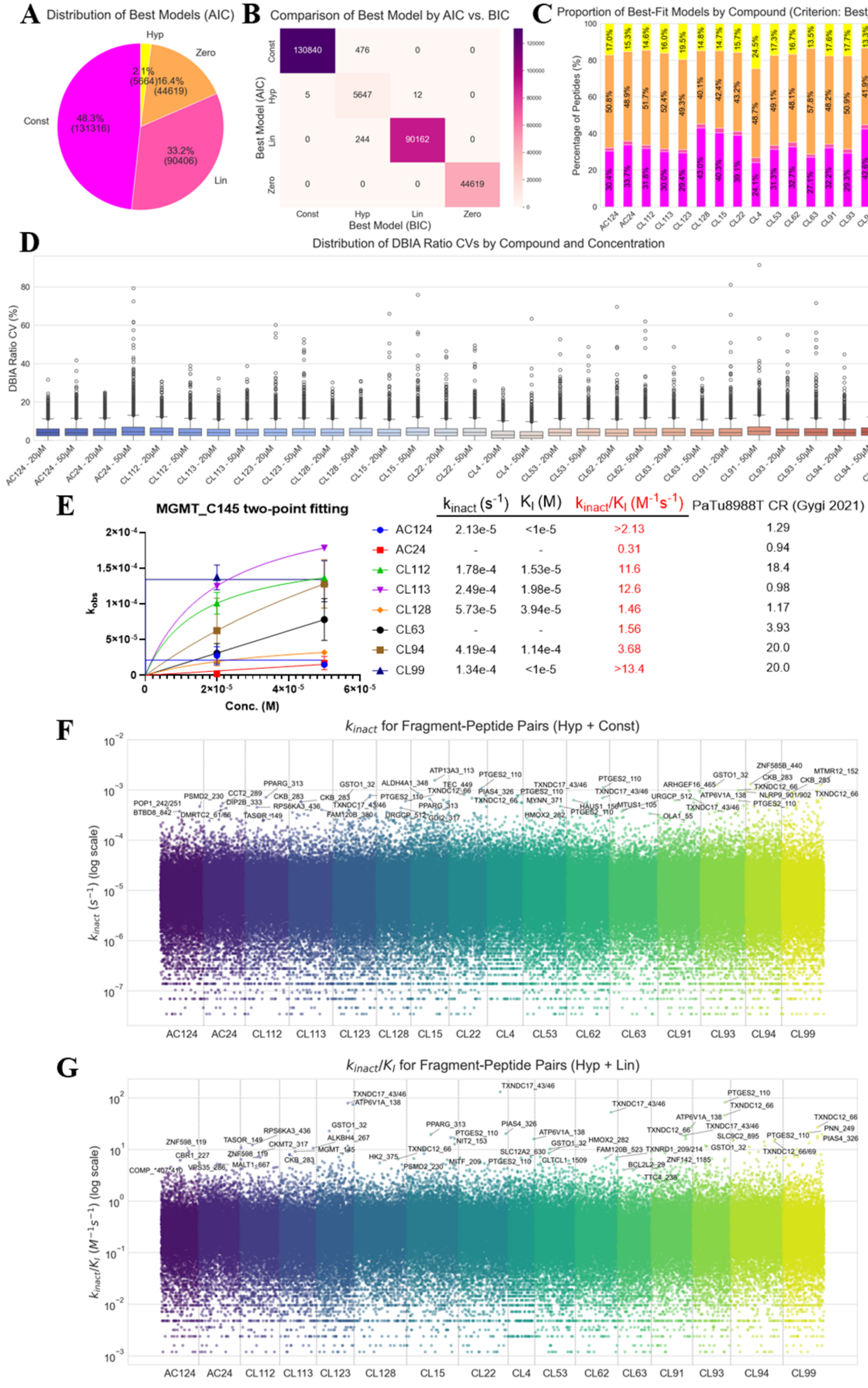

A Distribution of Best Models (AIC)
Hyp
Zero
Const
Lin
2.1% (5664)
16.4% (44619)
48.3% (131316)
33.2% (90406)
B Comparison of Best Model by AIC vs. BIC
Best Model (AIC)
Best Model (BIC)
Const Hyp Lin Zero
130840 476 0 0
5 5647 12 0
0 244 90162 0
0 0 0 44619
C Proportion of Best-Fit Models by Compound (Criterion: Best_Model_AIC)
Percentage of Peptides (%)
Lin
Hyp
Const
Zero
D Distribution of DBIA Ratio CVs by Compound and Concentration
DBIA Ratio CV (%)
E MGMT_C145 two-point fitting
kobs
Conc. (M)
kinact (s-1) | KI (M) | kinact/KI (M-1s-1) | PaTu8988T CR (Gygi 2021)
AC124 | 2.13e-5 | <1e-5 | >2.13 | 1.29
AC24 | - | - | 0.31 | 0.94
CL112 | 1.78e-4 | 1.53e-5 | 11.6 | 18.4
CL113 | 2.49e-4 | 1.98e-5 | 12.6 | 0.98
CL128 | 5.73e-5 | 3.94e-5 | 1.46 | 1.17
CL63 | - | - | 1.56 | 3.93
CL94 | 4.19e-4 | 1.14e-4 | 3.68 | 20.0
CL99 | 1.34e-4 | <1e-5 | >13.4 | 20.0
F kinact for Fragment-Peptide Pairs (Hyp + Const)
kinact (s-1) (log scale)
AC124 AC24 CL112 CL113 CL123 CL128 CL15 CL22 CL4 CL53 CL62 CL63 CL91 CL93 CL94 CL99
G kinact/KI for Fragment-Peptide Pairs (Hyp + Lin)
kinact/KI (M-1s-1) (log scale)
AC124 AC24 CL112 CL113 CL123 CL128 CL15 CL22 CL4 CL53 CL62 CL63 CL91 CL93 CL94 CL99

Figure 7. Applying two-point COOKIE-Pro strategy to profile binding kinetics for covalent fragment screening. (A) Pie chart showing the distribution of best-fit models (Zero, Constant, Linear, Hyperbolic) for all fragment-peptide pairs based on AIC. (B) Confusion matrix comparing the model selections made by AIC versus BIC. (C) Stacked bar chart illustrating the distribution of the four kinetic models for each of the 16 fragments screened. (D) Box plots showing the distribution of CVs of the DBIA ratios for each compound and concentration, indicating low variation among replicates. (E) A case study of two-point fitting for various fragments targeting the C145 residue of the MGMT protein. The plot shows the observed rate constant (k_{obs}) versus fragment concentration, with the table summarizing the calculated kinetic parameters (k_{inact}, K_I, k_{inact}/K_I) and literature competition ratio (CR) values. (F) Scatter plot displaying the landscape of inactivation rates (k_{inact}) for all peptides that fit the hyperbolic or constant models, grouped by fragment. (G) Scatter plot showing the landscape of inactivation efficiencies (k_{inact}/K_I) for all peptides that fit the hyperbolic or linear models, grouped by fragment.

Reviewer #2 (Remarks to the Author):

In this manuscript, Lin et al. developed the LC-MS-based COOKIE-Pro method which enables the determination of kinetic parameters of covalent inhibitors binding to proteins both on-target and off-targets in a proteome-wide fashion. The authors also argued that the model is broadly applicable for reactive cysteine profiling datasets by correlating the physical properties of the structures to their kinetic parameters. The manuscript is written in a very didactic manner making it easy to follow and understand. The authors showcase their method using the BTK inhibitors spebrutinib and ibrutinib and obtained on-par kinact/KI values with the literature.

While it was straightforward with spebrutinib, for ibrutinib the authors had to design and test serval probes to identify the one that would fit the requirement to accurately measure kinact/KI for the COOKIE-Pro method to work, making it difficult a one-fits-all approach with a generic probe like IAA. This example illustrates how important probe design is but presents a limitation of this method. While ibrutinib is less selective than spebrutinib, it is still a highly optimized drug molecule and therefore this raises doubt on the usage of COOKIE-Pro for compounds that are more promiscuous such as hit fragments from screening campaigns or natural product-based scaffolds. An example like that would

greatly strengthen the manuscript.

We appreciate the reviewer's thoughtful feedback and positive assessment of our manuscript. The reviewer raises two very important points: 1) the challenge of probe design, as illustrated by our work with ibrutinib, and 2) a resulting doubt about the method's applicability to more promiscuous compounds like fragments from a screening campaign. We agree that these are critical points to address.

Regarding the first point, the reviewer is correct that the optimization for the ibrutinib probe was non-trivial. This process, however, allowed us to establish several valuable "rules-of-thumb" for successful probe design, which we believe will be highly beneficial for future users of this method. These include the importance of maintaining the warhead's intrinsic reactivity, optimizing linker length, using appropriate probe concentrations to minimize non-specific labeling, and employing denaturing conditions to improve pulldown efficiency. While a generic probe like iodoacetamide is useful for peptide-level enrichment methods like SLC-ABPP, our protein-level pulldown approach intentionally uses a compound-derived probe. This is a crucial design feature that ensures we only pull down proteins that can bind the specific pharmacophore of interest, thus avoiding the enrichment of proteins that may have other reactive cysteines but are not true off-targets of the inhibitor itself.

Most importantly, to address the reviewer's second and primary concern, we have now included the exact type of experiment suggested. We sought to directly challenge the method with highly promiscuous molecules by performing a covalent fragment screen. We developed a high-throughput, "two-point COOKIE-Pro" strategy and applied it to a library of 16 fragments against the HeLa cell proteome.

This new study, detailed in the “Applying two-point COOKIE-Pro strategy to profile binding kinetics for covalent fragment screening” part of the manuscript and summarized in Figure 7, demonstrates that COOKIE-Pro is indeed well-suited for this application. The screen successfully generated a rich dataset of over 100,000 potential kinetic curves, allowing us to quantify the kinetic parameters (kinact and KI) for thousands of fragment-cysteine interactions. This allowed us to triage hits based on their kinetic profiles and decouple chemical reactivity from non-covalent affinity at scale, a key advantage for prioritizing fragment hits in a real-world drug discovery setting. We believe this new data strongly supports the broad utility of COOKIE-Pro and directly addresses the reviewer's concern, greatly strengthening the manuscript by demonstrating its effectiveness beyond highly optimized drug molecules.

Another drawback is the need of many different concentrations and time points. While the authors end the manuscript explaining this limitation and how to address it in theory (Figure 6), a real-life example would probably be a mix of these scenarios, different for each target. The number of technical replicates in this study is ambiguous. The authors should comment on how many replicates were performed for each data point and calculate CVs to demonstrate the reproducibility of the calculated kinetic parameters.

We thank the reviewer for raising these important points regarding the experimental throughput and the need for clear reporting on reproducibility. We acknowledge that the initial multi-point method is data-intensive, which is precisely why we developed the more streamlined two-point screening strategy to enhance throughput.

We agree that a purely theoretical discussion of the two-point method would be insufficient. The reviewer correctly anticipated that a real-life experiment would yield a mixture of kinetic scenarios. To address this, we have now included a large-scale, real-world application of this strategy by screening a library of 16 covalent fragments. Crucially, we did not assume a single kinetic model would fit all interactions. Instead, as detailed in the manuscript and shown in Figure 7, we applied an algorithmic approach using the Akaike Information Criterion (AIC) to classify each of the thousands of fragment-peptide interactions into the most appropriate kinetic model (linear, hyperbolic, or constant). This demonstrates that our triage scheme is not just a theoretical concept but a practical data analysis workflow that can handle the complex mixture of results from a real screening campaign.

Regarding the ambiguity in replicate numbers, we have clarified this throughout the manuscript. The spebrutinib validation was performed in three independent experiments, and the ibrutinib study was performed in two independent experiments, as stated in the respective figure legends. For the new two-point fragment screening, the experiment was conducted in two biological replicates, and each of those samples was injected twice as technical replicates for the LC-MS/MS analysis.

To explicitly demonstrate the reproducibility of our measurements, as requested, we have calculated the coefficients of variation (CVs) for the quantitative data from the fragment screen. As shown in the new Figure 7D, the data is highly precise, with the majority of competition ratios showing CVs of less than 10% between replicates. We have also included additional supplementary figures (Figure S7) that show correlation plots for the final calculated kinetic parameters between replicates, further demonstrating the robustness and reproducibility of the method.

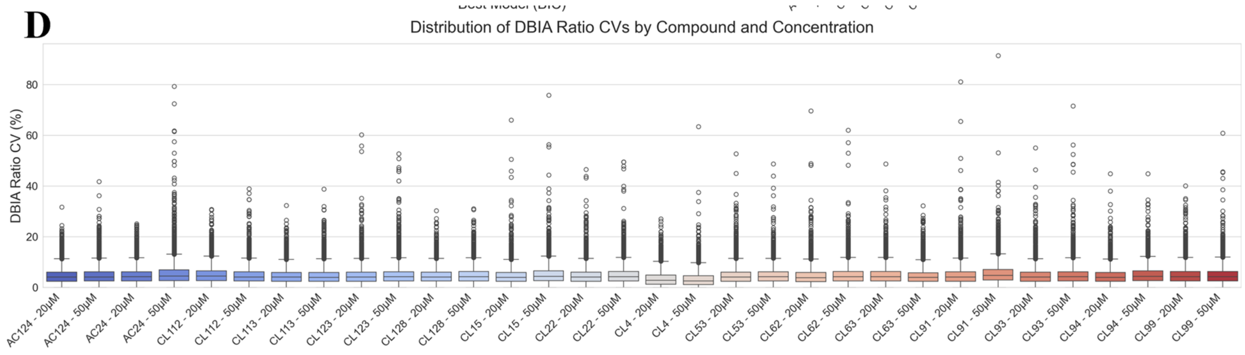

(Figure 7D) Box plots show the distribution of CVs of the DBIA ratios for each compound and concentration, indicating low variation among replicates.

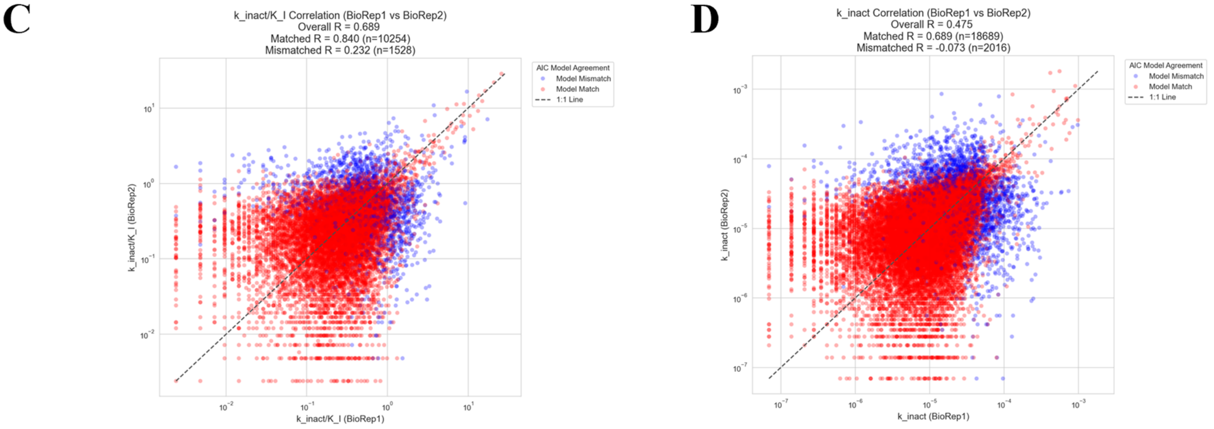

Figure S7(C) Correlation plot of calculated inactivation efficiencies (k_{inact}/K_I) between two biological replicates, colored by matched/unmatched model selection for replicates. (D) Correlation plot of calculated reactivity (k_{inact}) between two technical replicates, colored by matched/unmatched model selection for replicates.

Finally, as outlined at the beginning, the probe concentration used should saturate the targets but it is not possible to know when this is achieved unless the probe binds as potently as the unmodified inhibitor. This makes it impossible to use the COOKIE-Pro method as the first line of screening and requires extensive synthesis experience and time investment to measure kinact/KI compared to more traditional methods.

We thank the reviewer for this final point, which gets to the heart of the method's practicality. The concern that one must synthesize a perfectly optimized probe for every

compound, making the method impractical for first-line screening, is valid, and we realized this was a potential limitation. To address this, we have now included a large-scale, real-world fragment screening experiment that demonstrates a more pragmatic and high-throughput application of the COOKIE-Pro workflow.

While the deep profiling of a lead compound like ibrutinib benefits from a carefully optimized, potent probe, this is not a strict requirement for screening applications. In our new fragment screening study (Figure 7), we did not synthesize a custom probe for each of the 16 fragments. Instead, we used a generic, commercially available thiol-reactive probe, desthiobiotin-iodoacetamide (DBIA), for the "chase" step. This directly counters the assertion that extensive, bespoke synthesis is required for every compound and demonstrates that COOKIE-Pro can indeed be used as a first-line screening tool.

The logic of the "chase" step is not strictly about achieving thermodynamic saturation with a probe that is as potent as the inhibitor. Rather, the goal is to use a sufficiently high concentration of a reactive probe to rapidly and irreversibly occupy any cysteine sites that were not already covalently modified by the test compound in the first "pulse" incubation. By using the DBIA probe at a high concentration (500 μM), we effectively "quench" the reaction and capture a snapshot of the occupancy achieved by the test fragment.

Therefore, we have demonstrated two distinct use-cases for COOKIE-Pro:

1. **Deep Profiling:** For lead compounds like ibrutinib, investing time in synthesizing a specific, high-affinity probe allows for highly accurate kinetic measurements of on- and off-targets.
2. **High-Throughput Screening:** For initial screening of fragments, a generic reactive probe can be used in a "two-point" format to rapidly generate kinetic data on thousands of interactions at once, moving far beyond the single-point competition ratios offered by traditional methods.

We believe this new data demonstrates that the COOKIE-Pro framework is flexible and can be adapted to different stages of the drug discovery pipeline, overcoming the limitations the reviewer rightfully identified.

In light of all these uncertainties and limitations, I believe that this work is too premature to be published in Nat Commun.

We thank the reviewer for their rigorous and detailed feedback, which has prompted us to substantially revise and strengthen the manuscript. We believe that the initial concerns regarding the method's scalability, practicality for screening, and reproducibility have now

been fully addressed with significant new data.

Reviewer #3 (Remarks to the Author):

I co-reviewed this manuscript with one of the reviewers who provided the listed reports. This is part of the Nature Communications initiative to facilitate training in peer review and to provide appropriate recognition for Early Career Researchers who co-review manuscripts.

Reviewer #4 (Remarks to the Author):

Noteworthy Results:
- The development of COOKIE-Pro represents an advance in profiling covalent inhibitor binding kinetics across the proteome, addressing a key challenge in the field. I can see this technique being used to normalize competition ratios based on the intrinsic reactivity of the electrophile.
- The method successfully determined kinact and KI values for both intended targets and off-targets of BTK inhibitors
- The suggested compatibility with SLC-ABPP datasets substantially enhances the broader utility of this approach.

Methodology Soundness:
The experimental approach appears to be generally robust, with appropriate controls and validation. Some suggestions for improvement:
- Test the method with a broader range of covalent inhibitors beyond BTK inhibitors. In particular, the authors should validate one or more inhibitors found from SLC-ABPP, or equivalent proteome-wide screens to back up their assumptions about shared K_inact and that COOKIE-PRO is compatible with single-dose, single-timepoint SLC-ABPP. Most covalent chemoproteomics screens are not identifying compounds as selective or potent as the clinical compounds used in this study. While the authors suggest that the method is compatible with SLC-ABPP, without experimental validation there is no evidence to support this.

We thank the reviewer for their valuable feedback on the manuscript's methodology. We agree that a key test for our method's utility is its application to a broader range of compounds, particularly the less selective and potent hits that typically emerge from large-scale screening campaigns like SLC-ABPP. The reviewer's point that our claim of compatibility with SLC-ABPP requires direct experimental validation is well-taken.

To address this, we have performed a new study that is now included in the manuscript. We developed a high-throughput, "two-point" COOKIE-Pro strategy and used it to profile a library of 16 different covalent fragments representative of the types of hits found in chemoproteomic screens (please refer to our response to review 1).

This new experiment directly addresses the reviewer's suggestions:

1. **It tests a broader range of inhibitors**, moving beyond the highly optimized BTK inhibitors to less selective fragment-like molecules.

2. **It provides the requested experimental validation.** Instead of relying on the assumption of a shared kinact value for a given warhead class (as was done in our re-analysis of published single-point data), our new two-point experiment allows for the direct calculation of kinetic parameters (kinact and KI) for thousands of individual fragment-cysteine interactions. This provides robust, experimental evidence that the COOKIE-Pro framework is applicable to screening hits.

The results, presented in Figure 7, demonstrate that our method can successfully characterize the kinetic profiles of these less potent compounds, yielding inactivation efficiencies consistent with typical fragment-based hits. This new data provides the necessary evidence to support our claim that COOKIE-Pro is a valuable and compatible tool for deconvoluting the contributions of affinity and reactivity from large-scale screening data.

- Include error analysis for the two-point method to better understand its limitations. As above, the authors should validate a protein-ligand interaction from screening using the two-point method.

We thank the reviewer for the suggestions to include a more explicit error analysis and to validate a specific protein-ligand interaction from our screening data.

To address the first point, we have incorporated a multi-faceted error analysis for the two-point method. Our primary method for ensuring the quality of our kinetic fits is the use of the Akaike Information Criterion (AIC) and Bayesian Information Criterion (BIC) for model selection. These criteria, which are based on the residual sum of squares (RSS) of the fit, allow us to choose the most appropriate kinetic model for each of the thousands of

interactions while penalizing for model complexity to avoid overfitting. This provides a robust, quantitative, and automated quality control for each calculated parameter. Furthermore, we have also included a detailed discussion of the statistical limitations and potential sources of error for the two-point method in the manuscript's Discussion section.

To address the second point on validation, we present the case study of multiple fragments targeting the C145 residue of the MGMT protein, as shown in Figure 7E. We used our two-point COOKIE-Pro method to determine the kinetic parameters for several fragments against this single site. We then validated these findings by comparing them to orthogonal data from the previously published large-scale screening study by Kuljanin et al., 2021. Our analysis shows a strong concordance between the two datasets; for instance, fragments that we found to have very high inactivation efficiencies (kinact/KI) also had high competition ratios (CR) in the original screen. This successful cross-validation with a published, large-scale dataset provides strong evidence for the reliability of our two-point approach.

Data Analysis:
- Source code must be made available. Preferably, the code could be converted to Python or R, so that the analysis pipeline here could have the broadest impact.

Thank you for your suggestions. We deposit all the scripts and example data in Python at https://github.com/Hanfeng-Lin/COOKIE_scripts so that readers can reproduce the results and easily adopt into their pipeline.

- Line 722 states "high peptide confidence". What is the FDR cutoff (e.g. q-value <= 0.01), and at what level is the cutoff applied (e.g. PSM-level, peptide-level, protein-level)?

We clarify the statement as follows:

The results were filtered to include peptide spectrum matches (PSMs) with a high peptide confidence with PSM and peptide-level FDR<0.01 … Protein quantification uses both unique and razor peptides, with protein-level FDR <0.01 for strict and <0.05 for relaxed restrictions

----

- Line 725 of the manuscript states "Protein quantification uses both unique and random peptides" - I can only assume that 'random' is a typo.

It is indeed a typo, see corrected:

Protein quantification uses both unique and razor peptides.

- Include statistical analysis of reproducibility across biological replicates. Figs 3 and 4 should show biological replicates/error-bars.

We thank the reviewer for emphasizing the critical importance of demonstrating reproducibility with appropriate statistical analysis and clear data visualization.

We have ensured that the manuscript fully addresses these points. For the spebrutinib and ibrutinib studies, the kinetic plots in Figures 3 and 4 now display the data from our independent biological replicates. Each data point in the Michaelis-Menton plots (panels F in both figures) represents the mean value of the observed rate constant (kobs) across these replicates with error bars representing the standard error of the mean (SEM), as requested. The figure legends explicitly state that the results for spebrutinib are derived from three independent experiments and for ibrutinib from two independent experiments.

Furthermore, for our new two-point fragment screening study, we have included a detailed statistical analysis of its reproducibility. The quantitative data was highly precise, with coefficients of variation (CVs) below 10% for the majority of measurements, as shown in Figure 7D. We performed the screen in two biological replicates with two technical replicates. To demonstrate the reproducibility of our final calculated parameters, we have included correlation plots in the supplementary materials (Figure S7C, D) which show a high degree of correlation for the calculated parameters between these biological replicates.

We added a statistics section and a data reproducibility section in the method part to reflect the changes:

Statistics

For spebrutinib and ibrutinib COOKIE-Pro experiments, the k_obs obtained from each independent COOKIE-Pro experiment were combined as replicates to perform the Michaelis-Menton fitting. The reported standard error of k_inact and K_I was calculated by GraphPad 10.2 using symmetrical approximate confidence intervals.

Data reproducibility and code availability

COOKIE-Pro result for spebrutinib was reproduced in all three independent experiments. COOKIE-Pro result for ibrutinib was reproduced in all two independent experiments. Two-point COOKIE-Pro fragment screening was performed in two biological replicates, and each sample injected twice for LC-MS/MS analysis.

The mass spectrometry raw files for COOKIE-Pro quantitative multiplexed proteomics have been deposited in the MassIVE dataset under accession number MSV000095804. Python scripts for two-point COOKIE-Pro data analysis can be accessed through https://github.com/Hanfeng-Lin/COOKIE_scripts.

We believe these revisions and clarifications robustly demonstrate the statistical soundness and reproducibility of the COOKIE-Pro method.

Conclusion:
The work presented here is of good quality. Some proof-reading is necessary (typos, grammatical errors, such as on line 230). However, the addition of the SLC-ABPP compability & two-point screening seems almost like an afterthought given the absence of experimental data supporting the suggested compability. Given that compability with proteome-wide screening methods like SLC-ABPP would substantially enhance the utility of COOKIE-PRO, I think this should be addressed if the manuscript is to be published in Nature Communications. Without validation, this manuscript is essentially a technical note. Additionally, the absence of source code necessary to reproduce the analysis should prevent publication, given the central role of the analysis in this work.

We thank the reviewer for their positive assessment of our work's quality and for their constructive feedback, which has been invaluable in improving the manuscript. We have corrected the typos and grammatical errors as suggested. We also agree that the original manuscript's claims regarding compatibility with proteome-wide screening methods

required substantial experimental validation to elevate the work beyond a technical note. We are confident that we have now fully addressed these major concerns with significant new data and resources.
